# Identifying Biomarkers and Therapeutic Targets by Multiomic Analysis for HNSCC: Precision Medicine and Healthcare Management

**DOI:** 10.1021/acsomega.3c07206

**Published:** 2024-03-07

**Authors:** Hafeeda Kunhabdulla, Ram Manas, Ashok Kumar Shettihalli, Ch. Ram Mohan Reddy, Mohammed S. Mustak, Raghu Jetti, Riaz Abdulla, Divijendranatha Reddy Sirigiri, Deden Ramdan, Muhammad Imam Ammarullah

**Affiliations:** † Department of Oral Pathology, Yenepoya Dental College, Yenepoya (Deemed to be University), Deralakatte, Mangalore 575018, India; ‡ Department of Biotechnology, B.M.S. College of Engineering, Bull Temple Road, Bengaluru 560019, India; § Department of Computer Applications (MCA), B.M.S. College of Engineering, Bull Temple Road, Bengaluru 560019, India; ∥ Department of Applied Zoology, Mangalore University, Mangalagangothri 574199, Karnataka, India; ⊥ Department of Basic Medical Sciences, College of Applied Medical Sciences, King Khalid University, Abha 61421, Saudi Arabia; # Department of Management Science, Faculty of Social Science and Political Science, Universitas Pasundan, Bandung 40261, West Java, Indonesia; ∇ Department of Mechanics and Aerospace Engineering, College of Engineering, Southern University of Science and Technology, Shenzhen 518055, Guangdong, China; ○ UNDIP Biomechanics Engineering & Research Centre (UBM-ERC), Universitas Diponegoro, Semarang 50275, Central Java, Indonesia; ◆ Biomechanics and Biomedics Engineering Research Centre, Universitas Pasundan, Bandung 40153, West Java, Indonesia

## Abstract

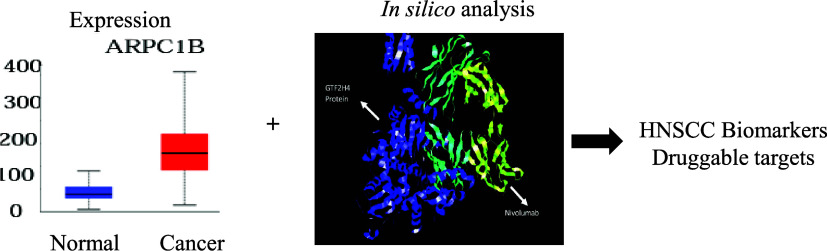

**Background:** Head and neck squamous cell carcinoma (HNSCC) is one of the major types of cancer, with 900,000 cases and over 400,000 deaths annually. It constitutes 3–4% of all cancers in Europe and western countries. As early diagnosis is the key to treating the disease, reliable biomarkers play an important role in the precision medicine of HNSCC. Despite treatments, the survival rate of cancer patients remains unchanged, and this is mainly due to the failure to detect the disease early. Thus, the objective of this study is to identify reliable biomarkers for head and neck cancers for better healthcare management. **Methods:** In this study, all available, curated human genes were screened for their expression against HNSCC TCGA patient samples using genomic and proteomic data by various bioinformatic approaches and datamining. Docking studies were performed using AutoDock or online virtual screening tools for identifying potential ligands. **Results:** Sixty genes were short-listed, and most of them show a consistently higher expression in head and neck patient samples at both the mRNA and the protein level. Irrespective of human papillomavirus (HPV) status, all of them show a higher expression in cancer samples. The higher expression of 30 genes shows adverse effects on patient survival. Out of the 60 genes, 12 genes have crystal structures and druggable potential. We show that genes such as GTF2H4, HAUS7, MSN, and MNDA could be targets of Pembrolizumab and Nivolumab, which are approved monoclonal antibodies for HNSCC. **Conclusion:** Sixty genes are identified as potential biomarkers for head and neck cancers based on their consistent and statistically significantly higher expression in patient samples. Four proteins have been identified as potential drug targets based on their crystal structure. However, the utility of these candidate genes has to be further tested using patient samples.

## 1 Introduction

Head and neck squamous cell carcinoma (HNSCC) is the most prevalent cancer that develops in the mucosal epithelium of the oral cavity, nasal cavity, pharynx, and larynx.^1^ The prevalence of HNSCC varies among different geographical areas and has been generally attributed to the use of tobacco, alcohol consumption, Epstein–Barr virus, human papillomavirus (HPV), Candidiasis, environmental factors, poor oral hygiene, and malnutrition.^2,3^ Long-term exposure to these carcinogens in the upper aerodigestive tract can result in dysplastic changes in the mucosal epithelium, ultimately resulting in cancer.^4^

Worldwide, 98,400 new cases and 48,100 oropharyngeal cancer deaths were reported in 2020, with age standardized rates (ASRs) of 1.1 and 0.51 per 100,000 for incidence and mortality, respectively.^5^ As per the American Cancer Society estimates, 54,000 new cases and 11,230 deaths were from oral cavity and oropharyngeal cancer by the year 2022, with the higher incidence coming from New Zealand, Australia, North America, and Europe. Mortality rate was the highest in the Caribbean, Central-East Europe, South Central Asia, Melanesia, and Western Europe. A recent study revealed that among the South Indian population, young adults are more likely than older people to use tobacco and chew on pans, which increases the risk of oral squamous cell carcinoma (OSCC), which is typically found in patients 45 years of age or younger.^6^ The incidence of HNC (head and neck cancer) continues to grow and is projected to increase by 30% (1.08 million new cases annually) by the year 2030.^7^

A recent publication by the Indian National Cancer Registry pointed out a difference in the oral cancer incident rates. Aizawl district of Mizoram has estimated 7 times more incident rates of cancer in males and 4 times in Osmanabad and in Maharashtra.^8^ Traditionally, surgical resections followed by postoperative chemoradiation is the major treatment for HNSCC, and this modality of treatment is still continuing. Low survival rates in HNSCC are mainly attributable to failure in early diagnosis.

The heterogeneity and relapsing nature of head and neck cancer require an improved understanding of the tumor nature in order to counteract the resistance, recurrence, and disparities in therapeutic response.^9^ The failure in early diagnosis is due to a lack of suitable diagnostic biomarkers for screening and diagnosis.^10^ Thus, new biomarkers for earlier detection as well as targeted therapies are essential for precision medicine.

Biomarkers are biological molecules, and their levels can be correlated with the presence and absence of specific diseases. These biomarkers also predict disease progression and can be used as potential targets for specific therapies. Druggability of target molecules that are causative agents of a disease is one of the desirable outcomes for many diseases, and it is much sought-after in the context of cancer. Biomarkers can also be attractive druggable targets especially if they happen to be proteins and are involved in the pathophysiology of the cancer. Tumor biomolecules are a new tool with which one can advance the early detection of cancer and improve monitoring, treatment, and survival. Currently, many types of biomarkers exist and can be measured during the changes in the host genome, protein expression profiles in metastasis, angiogenesis, and the presence of viral infection. In recent years, a handful of biomarkers are currently being studied and validated for clinical use in HNSCC detection, prognosis, and treatment.^11^ Furthermore, the ability to identify the differentially expressed genes (DEGs) in tumors using high-throughput methods like microarrays or ribonucleic acid (RNA) Seq technologies is well-established.^12^ Many gene expression profiles have been reported in HNSCC to encode the mRNA (mRNA) signatures and several DEGs.^13,14^

In the present study, the objective is to identify the differentially expressed genes in an unbiased way by screening RNAseq data of HNSCC patient samples from the TCGA (the cancer genome atlas) for 20,000 human genes. We also explored whether any of the protein biomarkers are druggable by screening against chemical ligands. Sixty genes were identified that show statistically significantly higher expression in HNSCC samples at the mRNA and protein levels. Further analysis showed that the higher expression of some of these genes leads to the poor survival of the affected individuals. Also, it identified a few genes that show a higher probability of being druggable. These candidate genes could be potential biomarkers and therapeutic targets for HNSCC, contributing to healthcare management, and from which patients are likely to benefit from specific therapies, thus having significant implications for resource allocation and treatment decisions.

## 2 Materials and Methods

### 2.1 Data Analysis

A list of around 42,000 annotated human genes was obtained from the HUGO gene nomenclature committee (HGNC).^15^ All of these genes were screened for their expression at mRNA and protein levels using the UALCAN web tool that profiles the expression of human genes from TCGA patient samples with level 3 RNAseq data. Here, RNAseq expression data for each gene was visualized by a box and whisker plot. Highcharts (Highsoft AS Highchart) were employed for visualization of the graphs. Usually, outliers were excluded from the graph. The “Statistics::T-Test” module from the Comprehensive Perl Archive Network (CPAN) was used in a PERL script to run the t-test.^16^ In the beginning, the mRNA expression profile was carried out, and genes were short-listed for their differential higher expression in head and neck cancers. Further, short-listed candidate genes were screened for their corresponding protein expression profile in the same head and neck cancer data set having proteomics data. Similarly, the mRNA expression profile for the short-listed candidate genes was carried out with respect to human papillomavirus (HPV) status and nodal metastasis.^17^

Protein–protein interaction and network for the short-listed candidate genes were carried out by using STRING.^18^ A list of genes was fed into the online string web tool for creating a protein–protein interaction network. Resulting images were downloaded, and publication-quality images were prepared.

### 2.2 Survival Analysis

SurvExpress was used to analyze the effect of gene expression on LUSC patient survival. This tool provides Kaplan–Meyer plots for the input genes and their corresponding *p*-values to determine their clinical significance.^19^ The quantize level option was used to transform gene expression values to specific levels. As an example, if three levels are used with uniform data (values from 0 to 1), all values lower than 0.333 were set to 0, values between 0.333 and 0.666 were set to 0.5, and values larger than 0.666 were set to 1.

### 2.3 Docking Studies

Computational simulation adopted docking methods in the present study with two types of docking studies, the first one was MTiOpenScreen, MTiAutoDock (which is based on AutoDock Vina)^20^ and the second one was Hex Dock.^21^ For both studies, the receptor/protein structures of three genes (HAUS7, GTF2H4, MNDA) were downloaded from the AlphaFold protein structure database^22,23^, and the structure for MSN was downloaded from the RCSB database.^24^ MTiOpenScreen preprocesses the protein structure (remove water and ligand molecules and add a polar hydrogen) using Autodock MGL tools if it is uploaded in the PDB format. The filters were applied based on Lipinski’s rule of 5, where the molecular weight was set to less than 500, HBA less than 10, HBD less than 5, and finally log *P* less than 5,^25^ and the Diverse-lib was selected, which is the collection of diverse chemical compounds. Out of the 84 results, the top four ligands with the highest binding affinity to the target protein were selected, and their drug-likeness was analyzed using various properties such as nRot, HBA, HBD, LogP, and TPSA.^26^

For other proteins for which MTiOpenScreen did not yield any results, further studies were performed by docking them with monoclonal antibodies, which were approved by FDA and are currently on sale.^27^ The structures of these drugs were obtained from the RCSB database, and the virtual screening was performed using Hex dock^28^ with the following parameters: correlation type: shape + electrostatics; grid dimension: 0.6; twist range: 360; distance range: 40; receptor range and ligand range both as 180; and the side chains were removed. The results were visualized in RasMol after removing hydrogen atoms and applying ribbon display and chain color for better visualization.^29,30^

### 2.4 Mutational Analysis

Mutational and gene amplification was carried out by using the cBioportal web tool having TCGA HNSCC data sets.^31,32^ All short-listed candidate genes were checked for amplification or mutations using the TCGA data set pertaining to head and neck cancer. Genes showing at least 3–4% amplifications or mutations were selected, corresponding images were downloaded, and publication-quality figures were generated.

## 3 Results

### 3.1 HNSCC Differentially Expressed Genes

**Figure 1:**
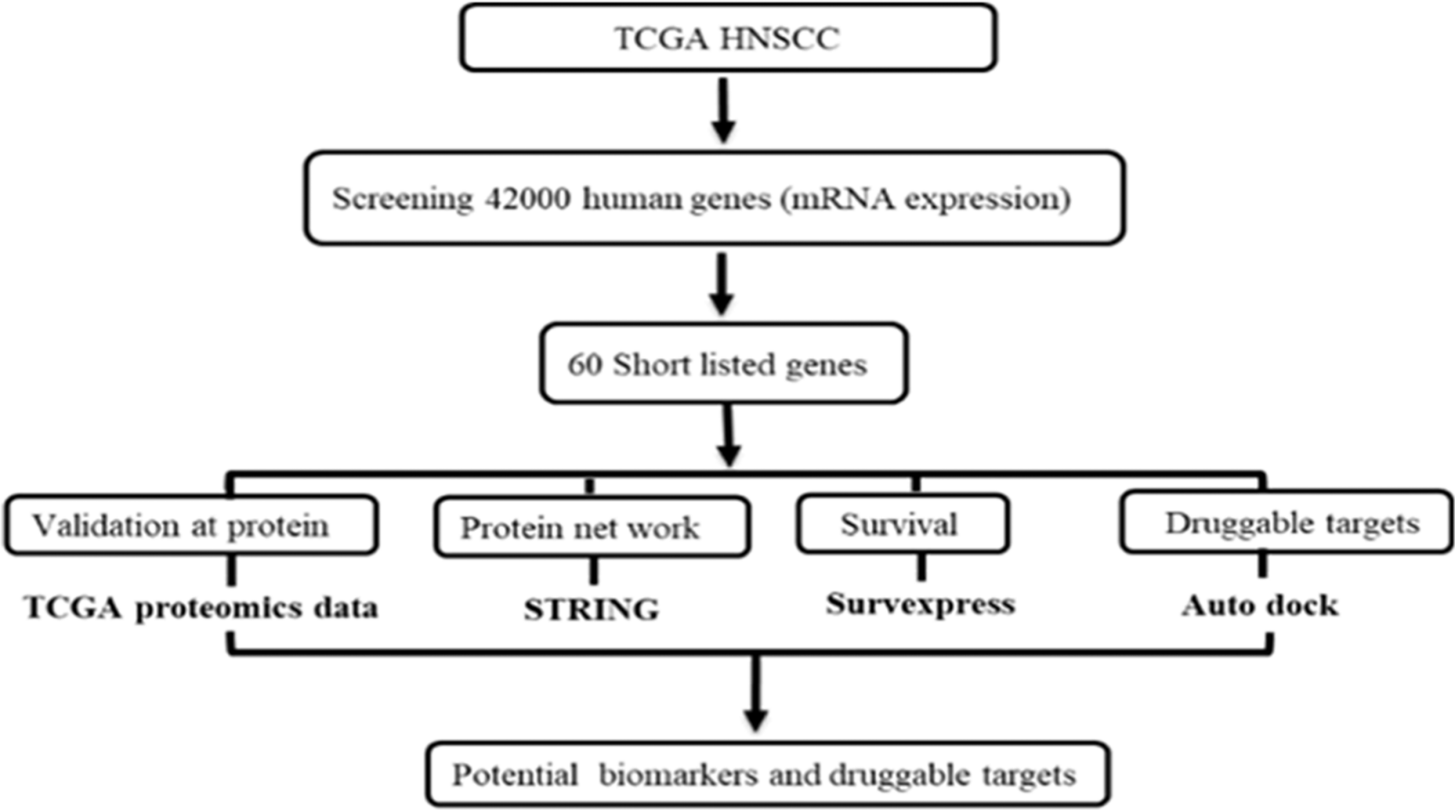
Flow diagram of the methodology followed.

In order to identify the differentially higher expressed genes in HNSCC patient samples, all curated human genes numbering around 42,000 were downloaded from the HGNC. The workflow diagram for the presentation of data analysis is depicted in Figure 1.

**Figure 2:**
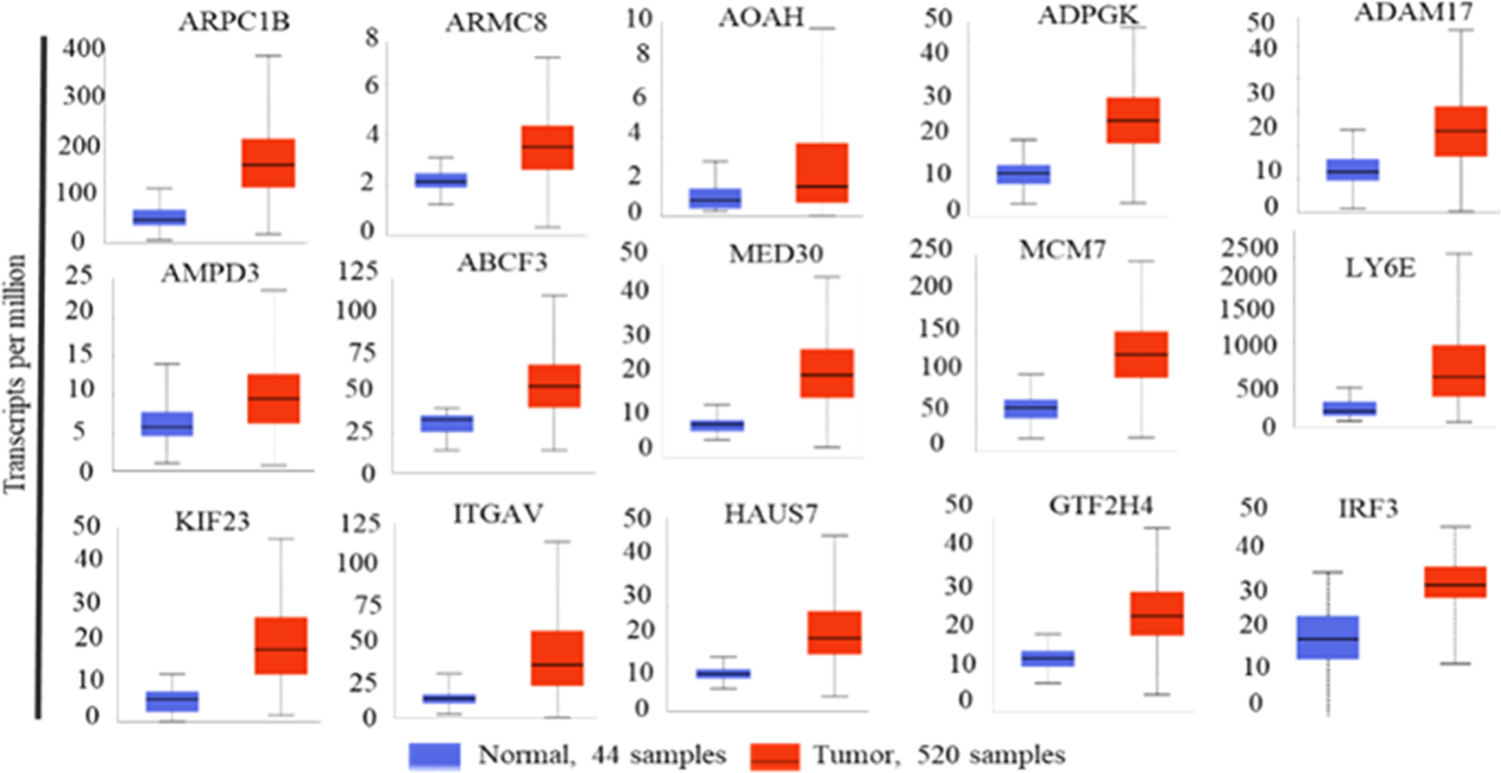
mRNA expression plots of head and neck cancer genes. N, normal sample with respective sample number; T, tumor sample with respective sample number.

All genes were screened for their expression against the TCGA RNAseq data pertaining to the HNSCC having a *p*-value of 10^–15^ or below to obtain statistically significant higher expressed genes in cancer samples. These studies resulted in the identification of 60 genes that show differential higher expression HNSCC samples (Figures 2 and S1–S3).

**Figure 3:**
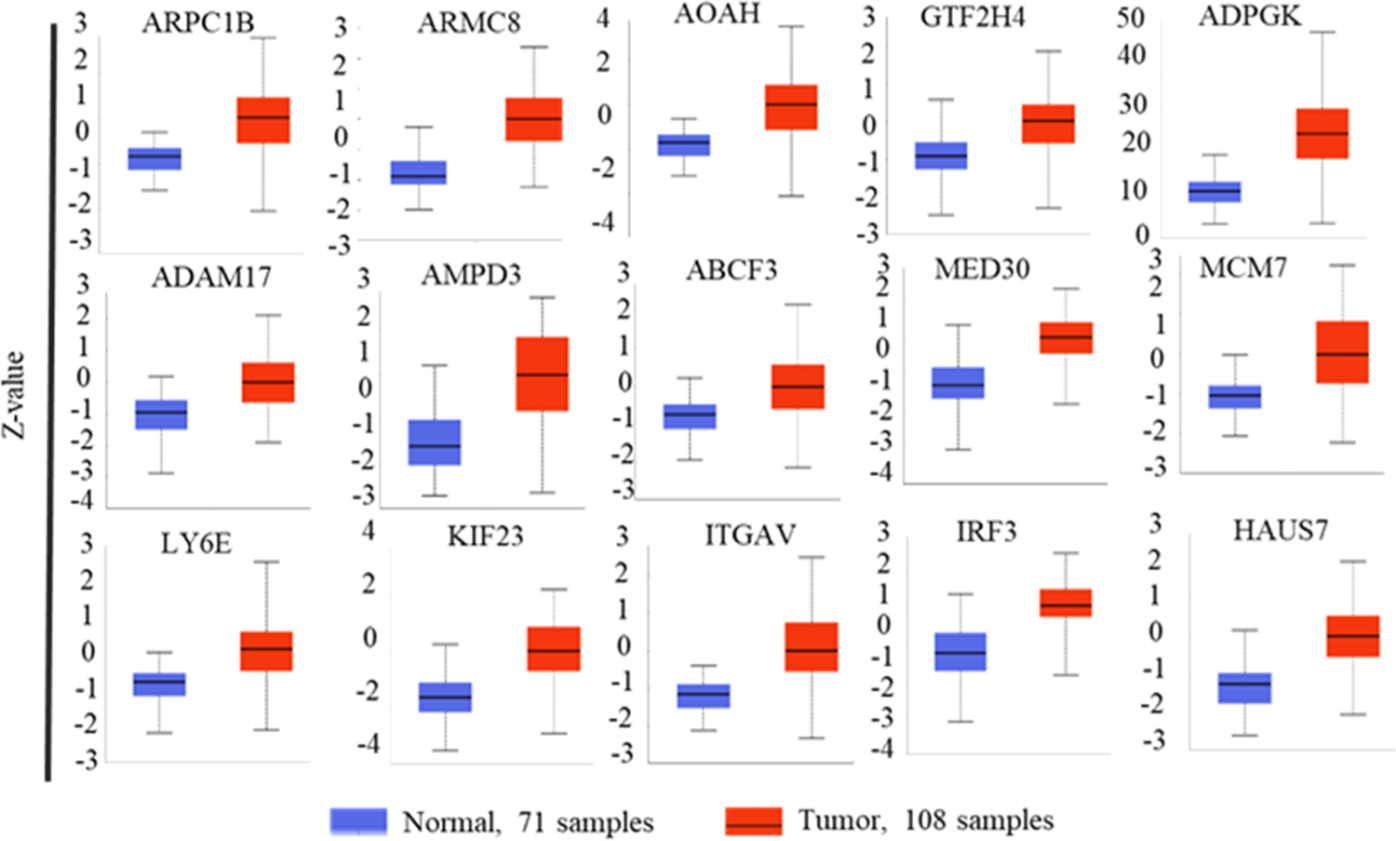
Protein expression plots of head and neck cancer genes.

**Figure 4:**
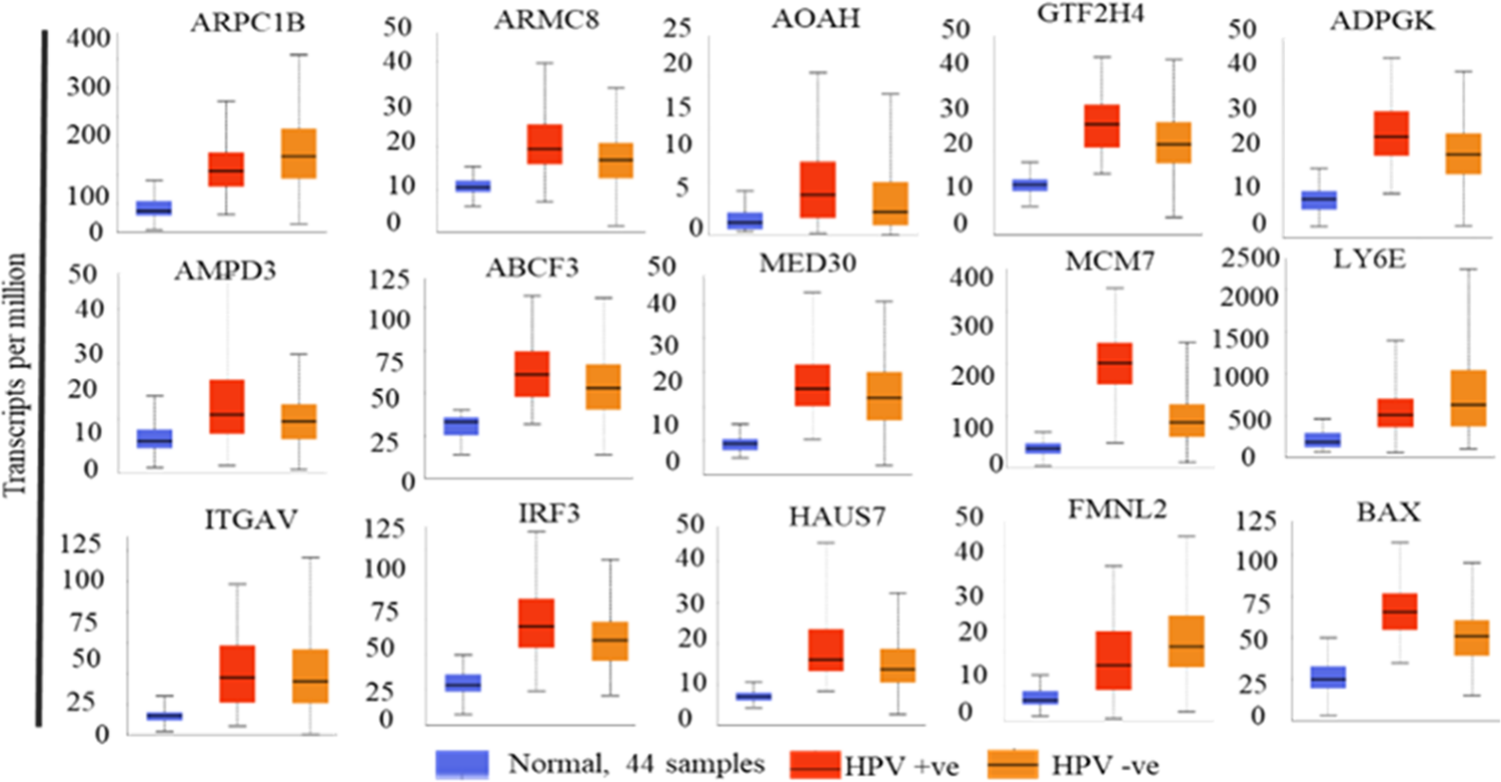
mRNA expression plots of head and neck cancer genes with respect to normal, HP positive, or negative HNSCC samples.

As the mRNA expression is one-dimensional and may not reflect automatic protein-level expression, in this study, we sought to determine the higher expression of these genes at the protein level as well in HNCC patient samples. As the proteomic data are available for some of the same patient samples in TCGA, we analyzed the protein expression for these genes. Results revealed that all 60 short-listed genes also show a higher protein expression in HNSCC samples, giving confidence that these genes show genuine differential higher expression (Figures 3 and S4–S6). As the hepatitis B virus is known to be associated with many subtypes of HNCC, we analyzed the expression of these genes in HPV-infected and noninfected samples. Data indicate that all of these genes show a higher expression irrespective of HPV status albeit with varying degrees of higher expression (Figures 4 and S7–S9).

**Figure 5:**
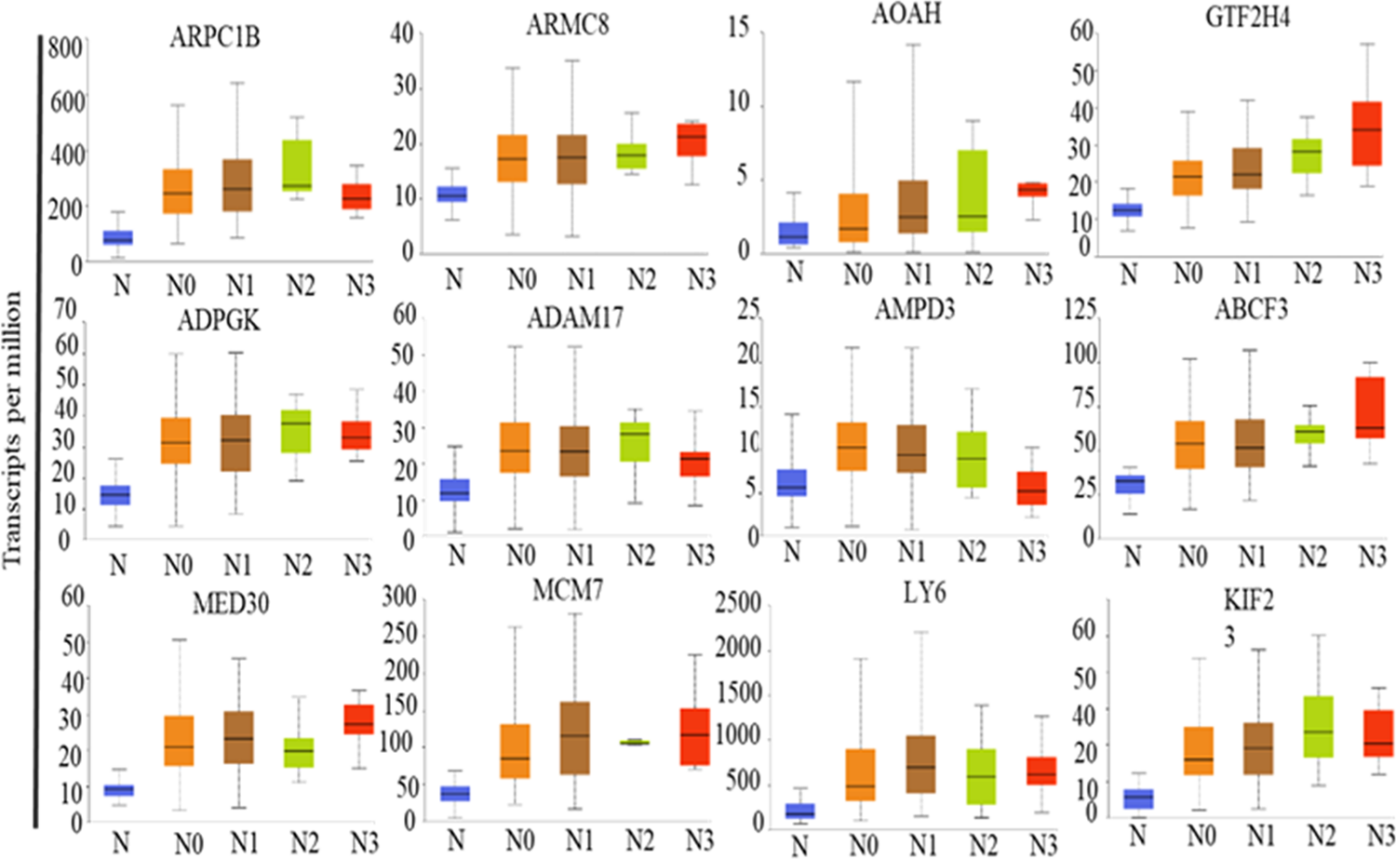
Nodal metastasis plots of head and neck cancer genes. N, normal (samples, i.e., *n* = 44); N0, no regional lymph node metastasis (*n* = 176); N1, metastases in 1–3 axillary lymph nodes (*n* = 67); N2, metastases in 4–9 axillary nodes (*n* = 12); N3, metastases in 10 or more axillary lymph nodes (*n* = 8).

In order to check whether these genes exhibit differential expression in the metastasis of HNCC, expression of these genes with respect to nodal status was assessed. All of these genes showed a higher expression in primary (N0) to metastasis (N1–N3), whereas very few genes showed a lower expression in N4 nodal status though still higher than in normal samples (Figures 5 and S10–S13). Overall, all of the short-listed genes show a higher expression with respect to primary cancer, HPV, and nodal status.

### 3.2 Mutational Analysis and Protein–Protein Interaction

**Figure 6:**
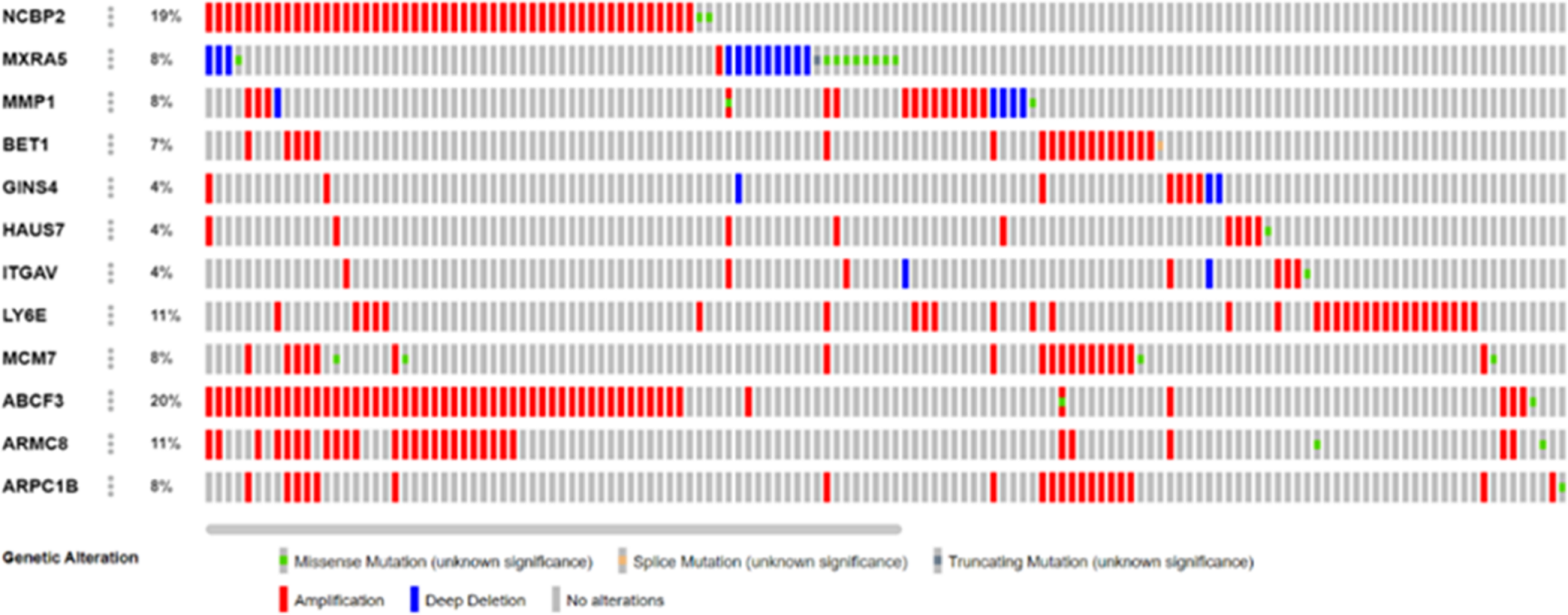
List of genes showing expression of more than 3%.

**Figure 7:**
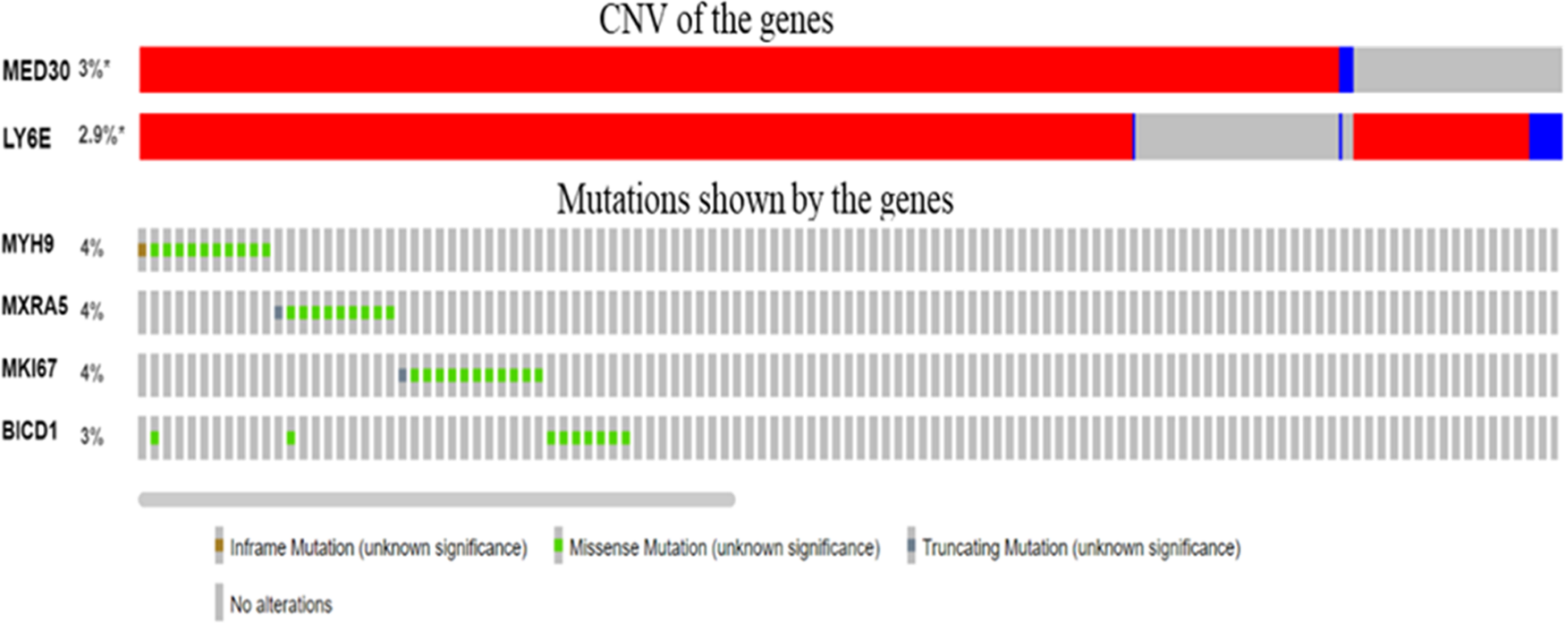
List of genes showing more than 3% mutations.

**Figure 8:**
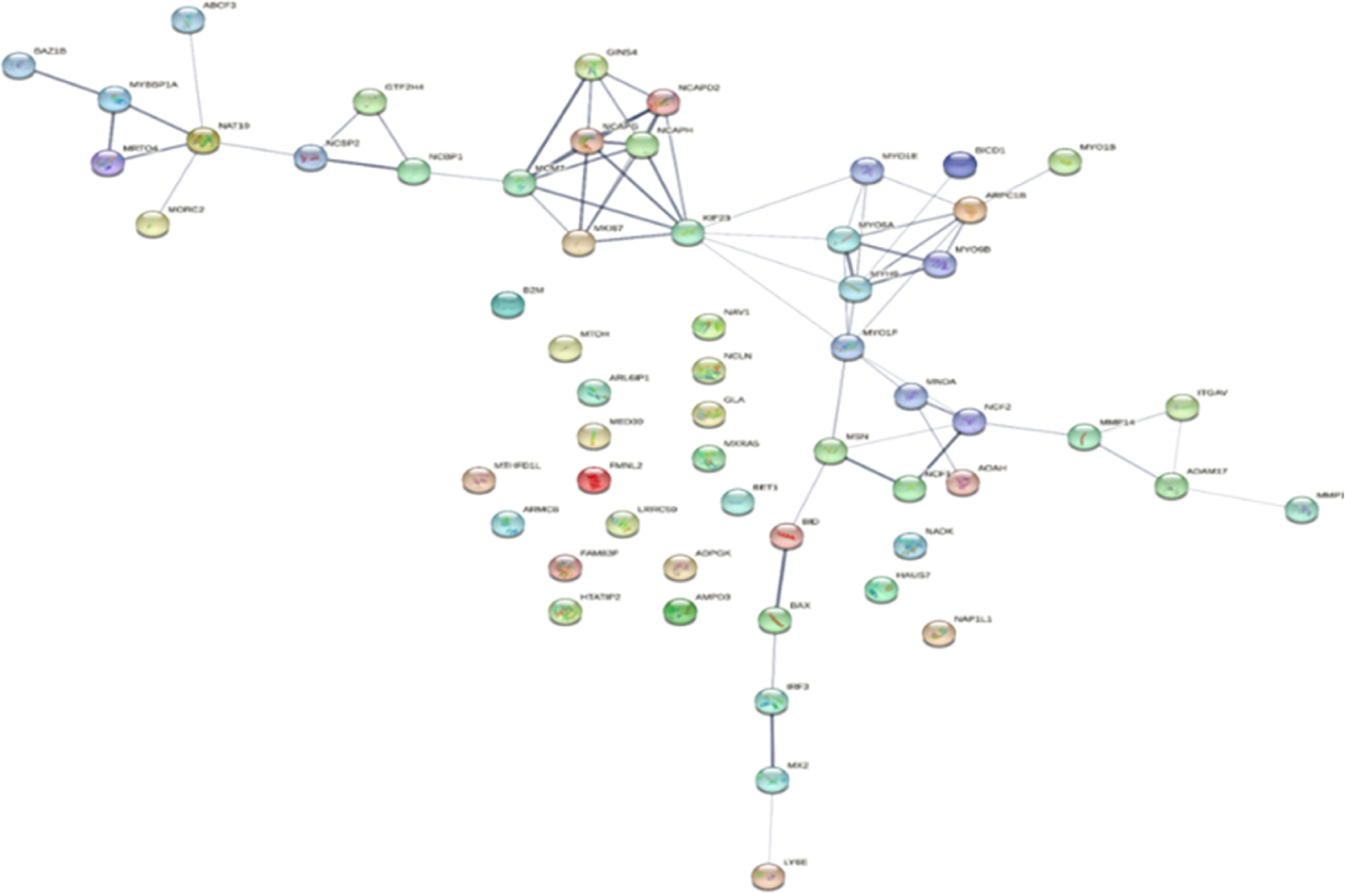
Protein–protein interaction network for the short-listed proteins.

It is a well-documented general phenomenon in many cancers that genes undergo mutations and amplifications, and here, we analyzed whether any of these genes show extensive mutations and amplifications. Very few genes such as MYH9, MXRAS, MK167, and BICD1 show mutations, with a frequency that is 4% or less (Figure 6). Similarly, very few genes show amplification but not to a significant level (Figure 7). To further assess whether these genes show any commonality in function or are associated with each other in any biological function, a network association among these proteins was explored. Results show that few proteins are interconnected, while most of them do not show any kind of interaction (Figure 8).

### 3.3 Higher Expression and Patient Survival

**Figure 9:**
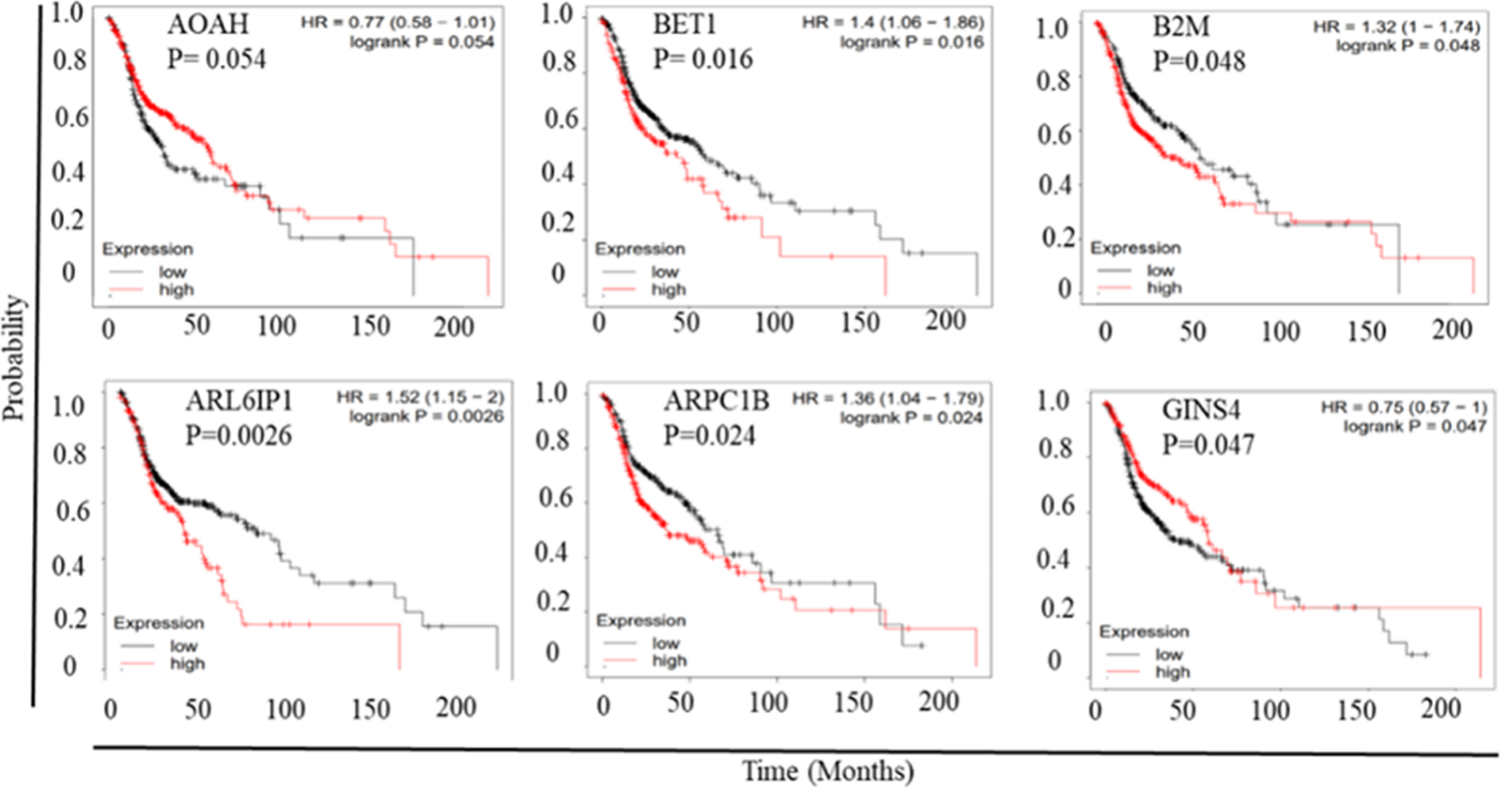
Kaplan–Meyer plots for survival based on short-listed gene expression.

As the short-listed candidate genes show consistently higher expression in HNSCC, we sought to determine whether the higher expression of these genes has any bearing on patient survival. Among the 60 short-listed genes, the higher expression of 30 genes shows adverse effects on patient survival with a *p*-value less than 0.05 (Figures 9 and S14–S17), while the higher expression of a few genes does relate to poor survival of the patients but is not statistically significant (data not shown).

### 3.4 Druggable Target Proteins

**Figure 10:**
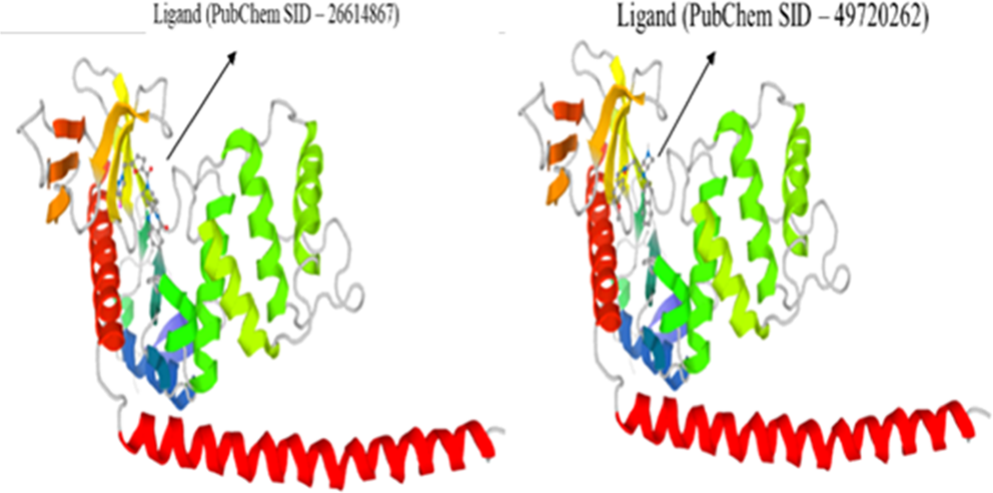
Binding of ligands to the MSN protein. This figure represents the ligands with binding affinity to the MSN protein column as mentioned in the PubChem SID. The binding energy is in kcal/mol. nRot, number of rotatable bonds; HBA, hydrogen-bond acceptor; HBD, hydrogen-bond donor; log *P*, partition coefficient; and MW, molecular weight of each compound. The topological polar surface area (TPSA) of a molecule is defined as the surface sum over all polar atoms or molecules, primarily oxygen and nitrogen, also including their attached hydrogen atoms.

**Figure 11:**
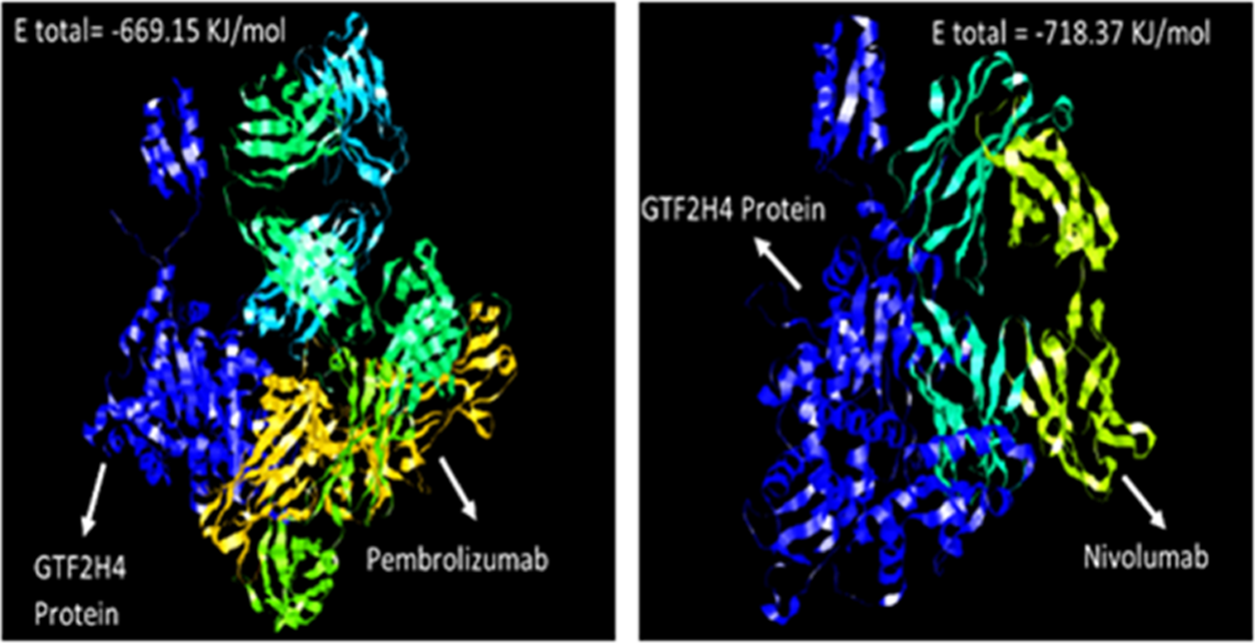
Binding of monoclonal antibodies to GTF2H4.

Out of the 60 short-listed genes, 14 proteins have crystal structures available either alone or in complex with bound ligands. We analyzed this to see if any of these proteins are druggable. Preliminary assessment using the cansar web tool^33^ identified that four proteins with a crystal structure may have a high probability of ligand/drug binding. For these four proteins, we analyzed binding affinities by virtual screening to identify highly probable ligands with a low binding free energy. Out of the four proteins analyzed for ligand binding by virtual screening, only the MSN protein is the target for ligands from the PubChem database. As shown in Figures 10 and S18, ligands such as SID–26614867, SID–49720262, SID–3711570, and SID–24840422 show a high binding affinity to MSN. Further studies were conducted to explore the binding of any of these proteins to the approved monoclonal antibodies for HNSCC. As shown in Figure 11, Pembrolizumab and Nivolumab show high affinity for binding to GTF2H4. Pembrolizumab and Nivolumab also bind to HAUS7, MSN, and MNDA proteins with good affinity (Figures S19–S21).

However, as monoclonal antibodies are designed to target a specific target protein, the binding monoclonal antibody to these proteins has to be verified experimentally.

## 4 Discussion

HNSCC is one of the most common types of cancer, with predominant cases coming from Asia.^2^ Early detection of the disease provides better chances of managing the disease with precision medicine, which can contribute to the healthcare management of HNSCC. However, in the majority of the cases, by the time of diagnosis, the disease is mostly in the advanced stage and provides little opportunity for treatment of the disease. In this context, suitable biomarkers for the accurate detection and diagnosis of HNSCC are very sought-after, and intensive efforts are ongoing. A recent study showed that lipid metabolism-related proteins, which are differentially expressed in OSCC with variable differentiation, can be used as prognostic biomarkers for OSCC.^34^ In this study, using very stringent criteria, we identified a list of genes that show higher differential expression in HNSCC using actual patient data samples that were profiled using a standard pipeline of gene expression studies at the mRNA and protein levels. Although many genes show a higher expression in HNSCC, we narrowed this down to 60 candidate genes and tested their expression at the mRNA and protein levels. The data presented here are statistically significant and were derived from actual patient samples, instilling high confidence in their potential utility as potential biomarkers for HNSCC. It would be interesting to know if any of these genes or proteins are known to be biomarkers or therapeutic candidates for other cancers and HNSCC in particular. A literature search resulted in the identification of 19 genes for the first time in this study as biomarkers or therapeutic targets for HNSCC and are also known to be studied for other cancers (Table 1). Only HAUS7 (Table S1) has been identified in this study for the first time as a biomarker and therapeutic candidate, and the remaining genes have been identified as either biomarkers or therapeutic candidates for HNSCC or other cancers from previous reports (Table S2). Over a period of time, many molecular biomarkers related to genomics, proteomics, and noncoding RNAs have been identified^35,36^, and many of them are currently undergoing validation. For routine diagnosis of the disease, cytokeratin 5, cytokeratin 6, and p63 are being used as biopsy staining markers.^37,38^ However, these markers are not suitable for screening or early identification of the disease. In this direction, the candidate genes and proteins identified in this study could be checked for their expression using early-stage cancer samples and their utility as reliable biomarkers or therapeutic candidates could be validated for the early screening and diagnosis of HNSCC. One limitation of this study is that since HNSCC is an amalgam of different cancers affecting various anatomical parts of the head and neck, whether these identified biomarkers are fit for all types of HNSCC detection is yet to be determined. However, it would be interesting to check if any or some of these identified biomarkers show consistently higher expression across all HNCSCC subtypes. A further analysis with different types of HNSCC samples for these candidate genes would be desirable to check their suitability as biomarkers, and therapeutic candidates are very much warranted.

**Table 1:** List of Genes Identified in This Study and Not Identified in Any Other Previous Research for Head and Neck Cancer but Were Identified in Other Types of Cancers

genes identified in this study	was it previously identified in HNC in other studies	other types of cancers it was previously studied in
ARMC8	no	lung, colon, and ovarian cancer
AOAH	no	breast and gastric cancer
GTF2H4	no	lung and cervical cancer
ADPGK	no	colorectal and breast
ABCF3	no	ovarian and oral cancer
MED30	no	gastric and bladder cancer
FMNL2	no	colorectal and breast cancer
ARL6IP1	no	cervical cancer
BAZ1B	no	colorectal and breast cancer
FAM83F	no	thyroid and lung cancer
BICD1	no	liver (hepatocellular) cancer and glioma
BET1	no	prostate and colorectal cancer
MNDA	no	lymphoma
MSN	no	breast cancer
MX2	no	prostate cancer and glioma
MXRA5	no	colorectal cancer, gastric cancer, and glioma
NADK	no	pancreatic cancer
NCAPD2	no	colorectal and breast cancer
NCBP1	no	liver (hepatocellular) and lung cancer

## 5 Conclusions

In this study, HNCC patient sample genomics and proteomics data were used to screen all known human genes for their differential expression in normal and HNCC cancer samples. Sixty genes were found to be highly expressed in cancer samples with statistical significance at the mRNA level. Out of the 60 genes, 19 genes are being identified as potential biomarkers for the first time in the context of HNCC. Monoclonal antibodies such as Pembrolizumab and Nivolumab show a high affinity for binding to GTF2H4. Many chemical ligands such as SID–26614867, SID–49720262, SID–3711570, and SID–24840422 show a high binding affinity toward the MSN protein, making it a potential therapeutic target in the context of HNCC, which can lead to improvements in precision medicine and healthcare management.

## Data Availability

Patient sample data sets presented in this study are available in the online TCGA portal and other sources as mentioned in Section 2.

## Supporting Information Available

mRNA and protein expression graphs, survival plots, protein–ligand docking data, and two tables of biomarkers for HNCC (PDF)
