# Supplementary material for: Identifying Biomarkers and Therapeutic Targets by Multiomic Analysis for HNSCC: Precision Medicine and Healthcare Management
Source: ACS Omega. 2024 Mar 7;9(11):12602–10. doi: 10.1021/acsomega.3c07206 (PMC10956120; doi:10.1021/acsomega.3c07206)
Supplement: Supplementary file 1 — ao3c07206_si_001.pdf [file ao3c07206_si_001.pdf]

Identifying Biomarkers and therapeutic targets by multi-omic analysis for HNSCC: Precision Medicine and Healthcare Management

Hafeeda Kunhabdulla[a], Ram Manas[b], Ashok kumar Shettihalli[b], Ch. Ram Mohan Reddy[c], Mohammed S Mustak[d], Raghu Jetti[e], Riaz Abdulla[a], Divijendranatha Reddy Sirigiri[b*], Deden Ramdan[f*], Muhammad Imam Ammarullah[g,h,i]

[a]Department of Oral Pathology, Yenepoya Dental College, Yenepoya (Deemed to be University), Deralakatte, Mangalore-575018, India.

[b]Department of Biotechnology, B.M.S. College of Engineering, Bull temple road, Bengaluru,

India.

[c]Department of Computer Applications (MCA), B.M.S. College of Engineering, Bull temple road, Bengaluru, India.

[d]Department of Applied Zoology, Mangalore University, Mangalagangothri-574199, Karnataka, India.

[e]Department of Basic Medical Sciences, College of Applied Medical Sciences, King Khalid

University, Abha, Saudi Arabia. Email: imahmood@kku.edu.sa

[f]Department of Management Science, Faculty of Social Science and Political Science,

Universitas Pasundan, Bandung 40261, West Java, Indonesia

[g]Department of Mechanics and Aerospace Engineering, College of Engineering, Southern University of Science and Technology, Shenzhen 518055, Guangdong, China; imamammarullah@gmail.com; https://orcid.org/0000-0002-8845-7202

[h]Undip Biomechanics Engineering & Research Centre (UBM-ERC), Universitas Diponegoro, Semarang 50275, Central Java, Indonesia

[i]Biomechanics and Biomedics Engineering Research Centre, Universitas Pasundan, Bandung 40153, West Java, Indonesia

*Correspondence: sdnreddy@gmail.com, deden.ramdan@unpas.ac.id

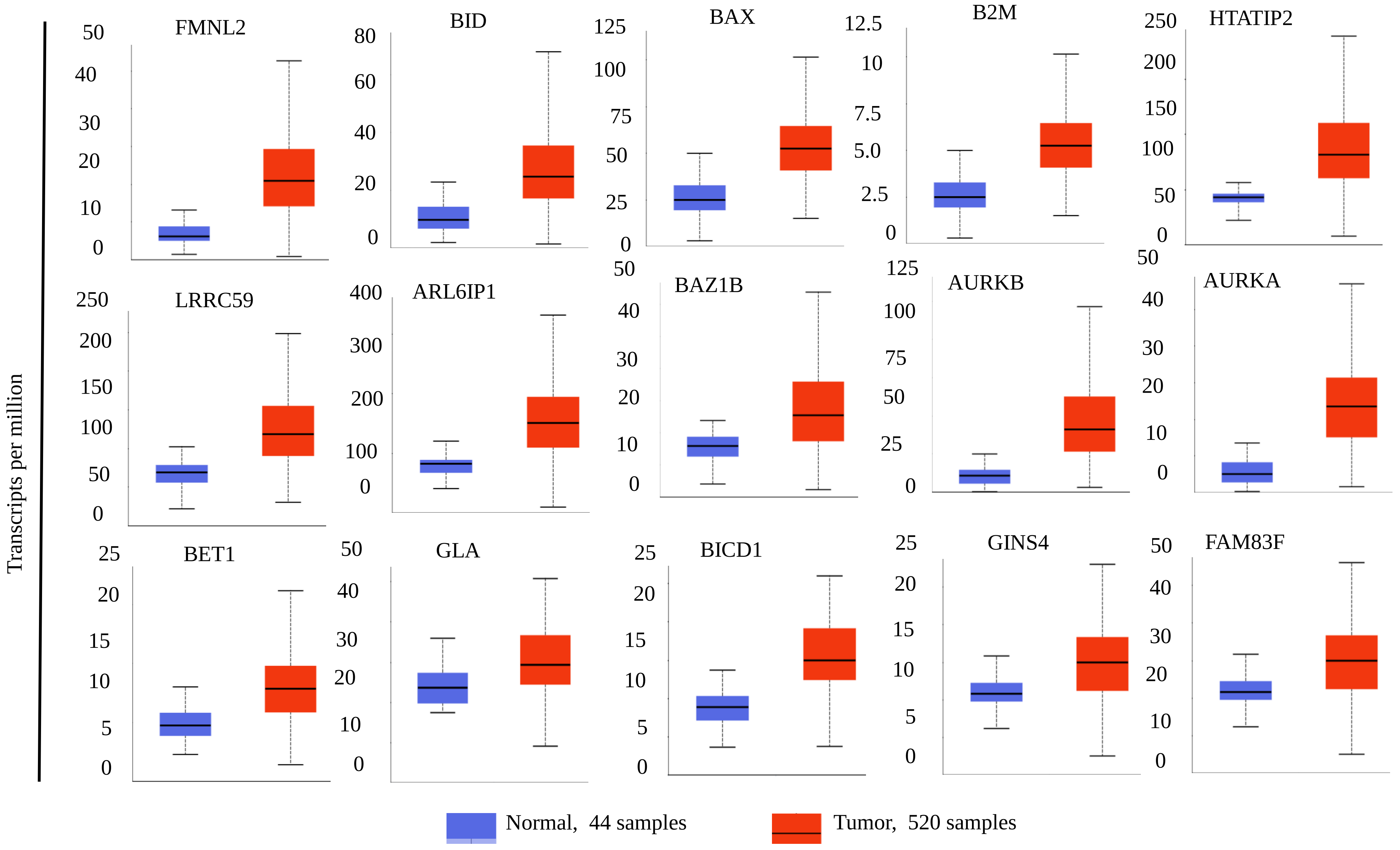

Figure S1: mRNA expression plots of Head and Neck cancer genes. N =Normal sample with respective sample number , T= tumor sample with respective sample number

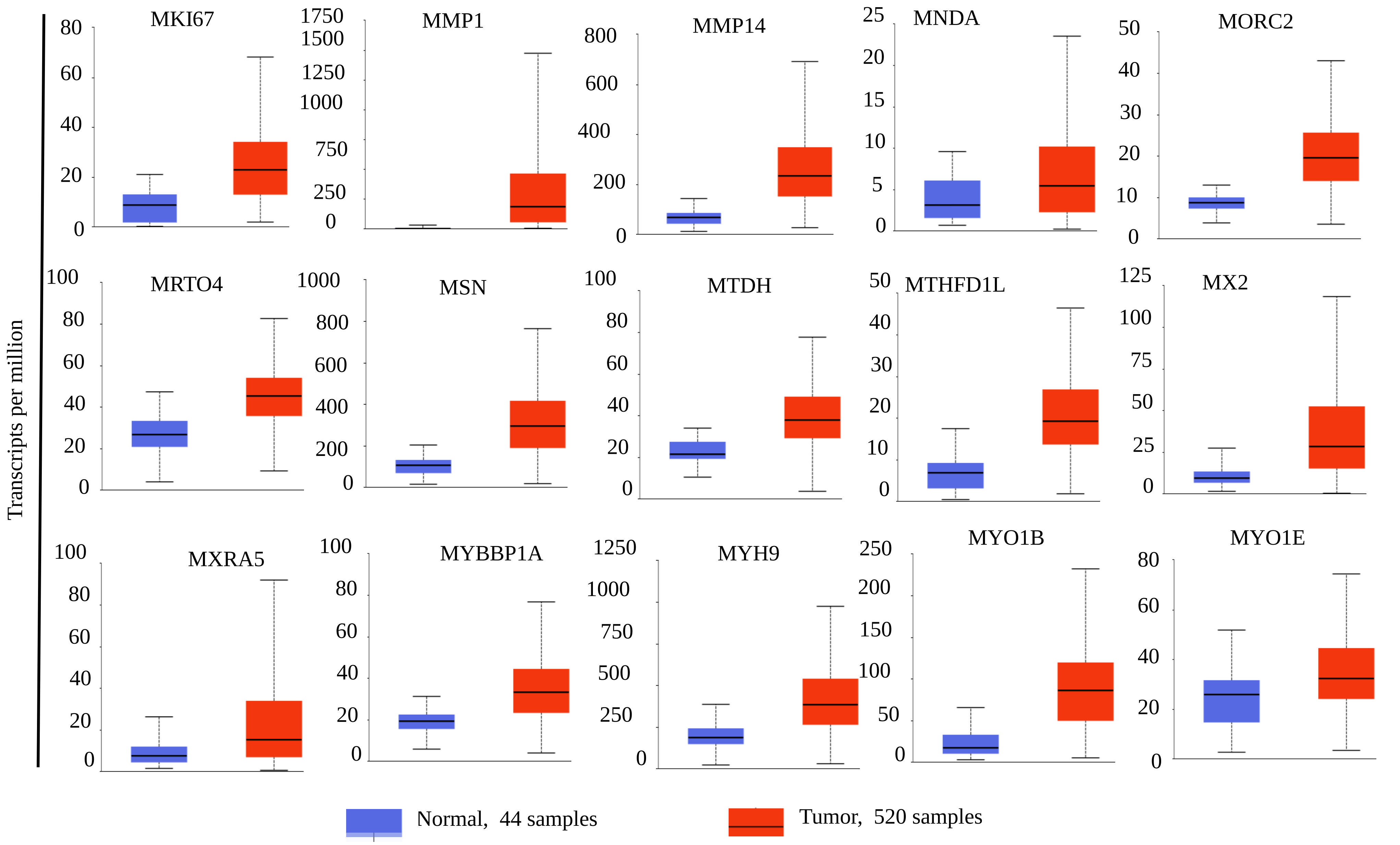

Figure S2: mRNA expression plots of Head and Neck cancer genes. N =Normal sample with respective sample number , T= tumor sample with respective sample number

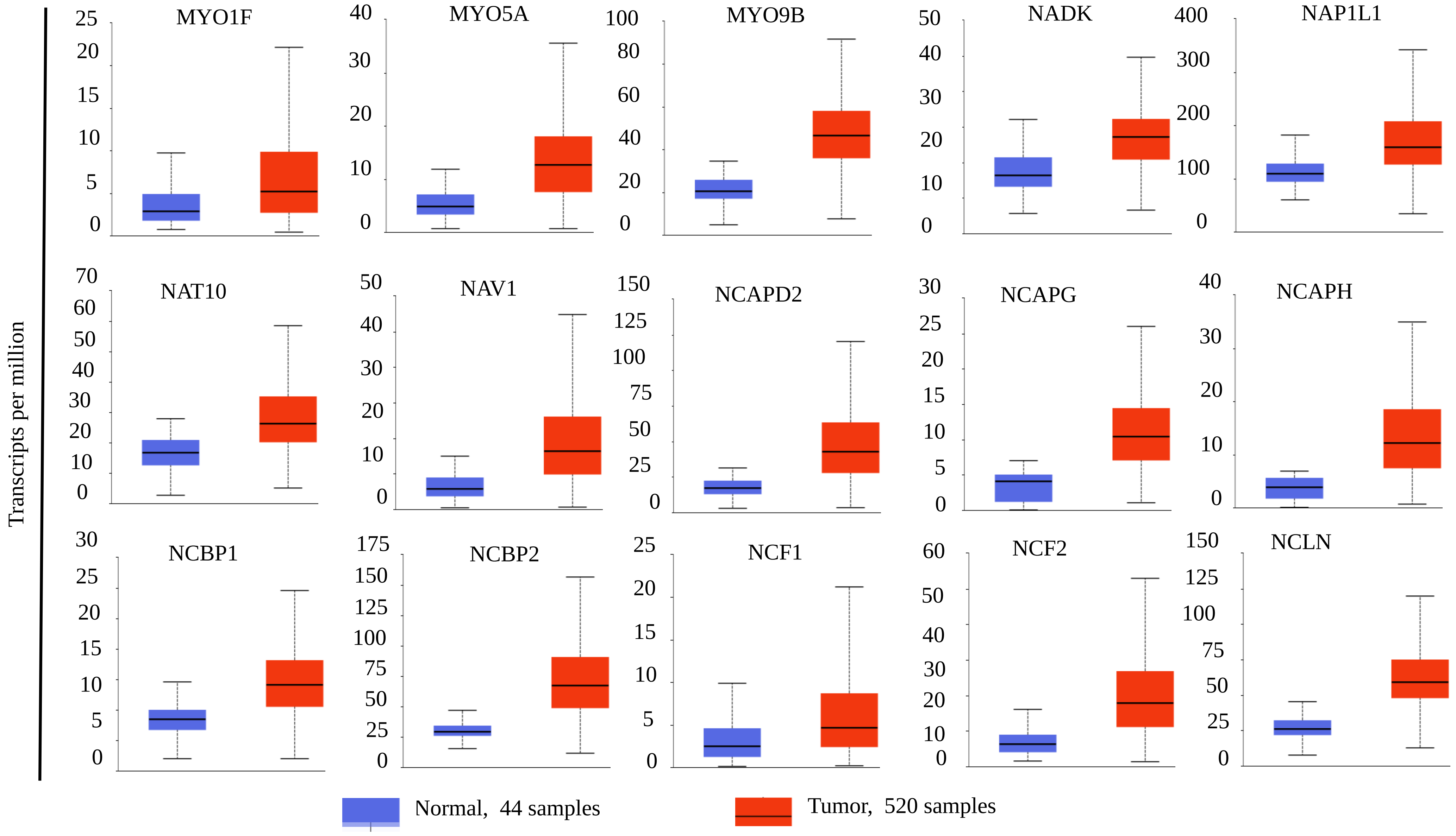

Figure S3: mRNA expression plots of Head and Neck cancer genes. N =Normal sample with respective sample number , T= tumor sample with respective sample number

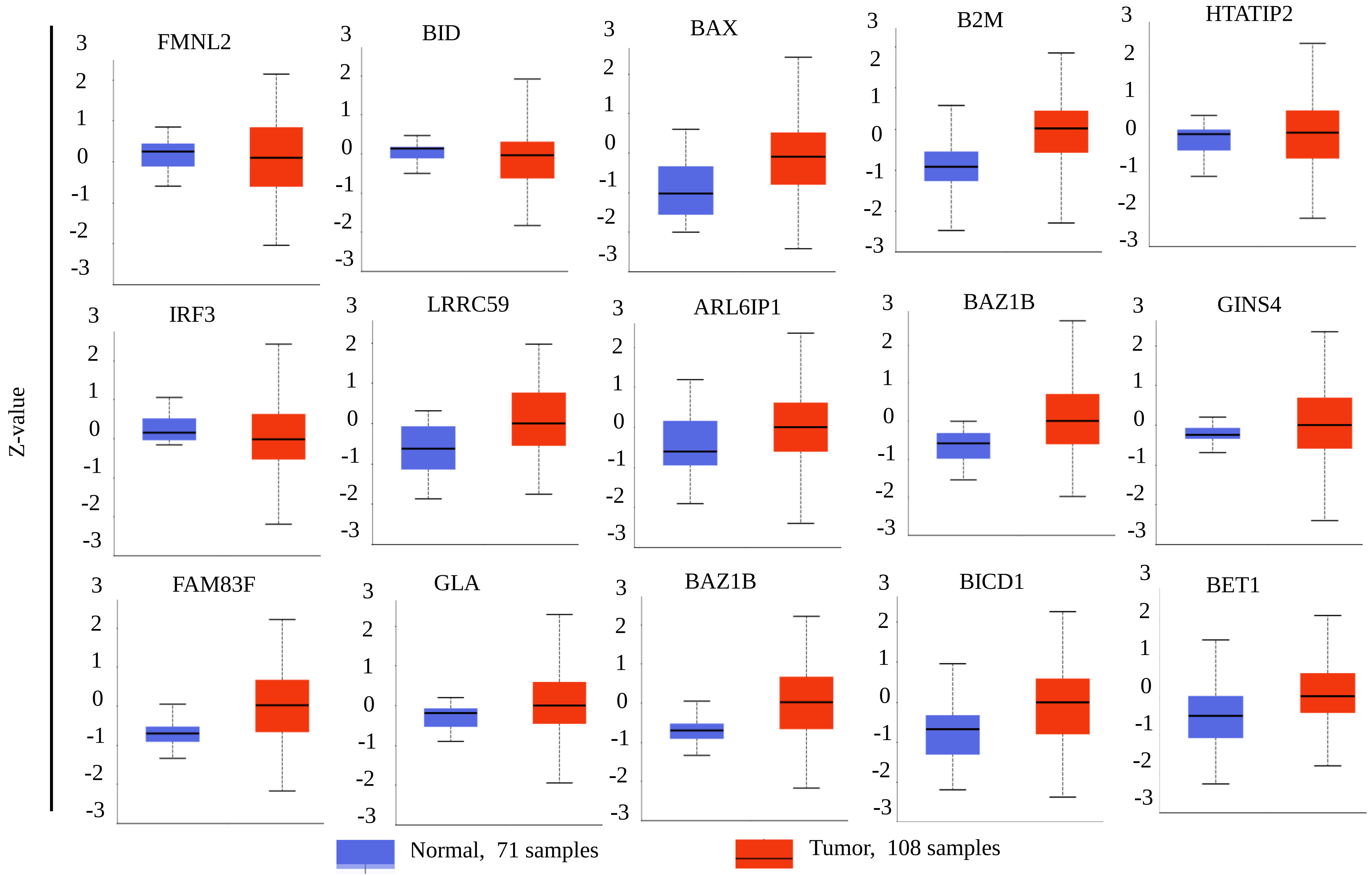

Figure S4: protein expression plots of Head and Neck cancer genes. N =Normal sample with respective sample number , T= tumor sample with respective sample number

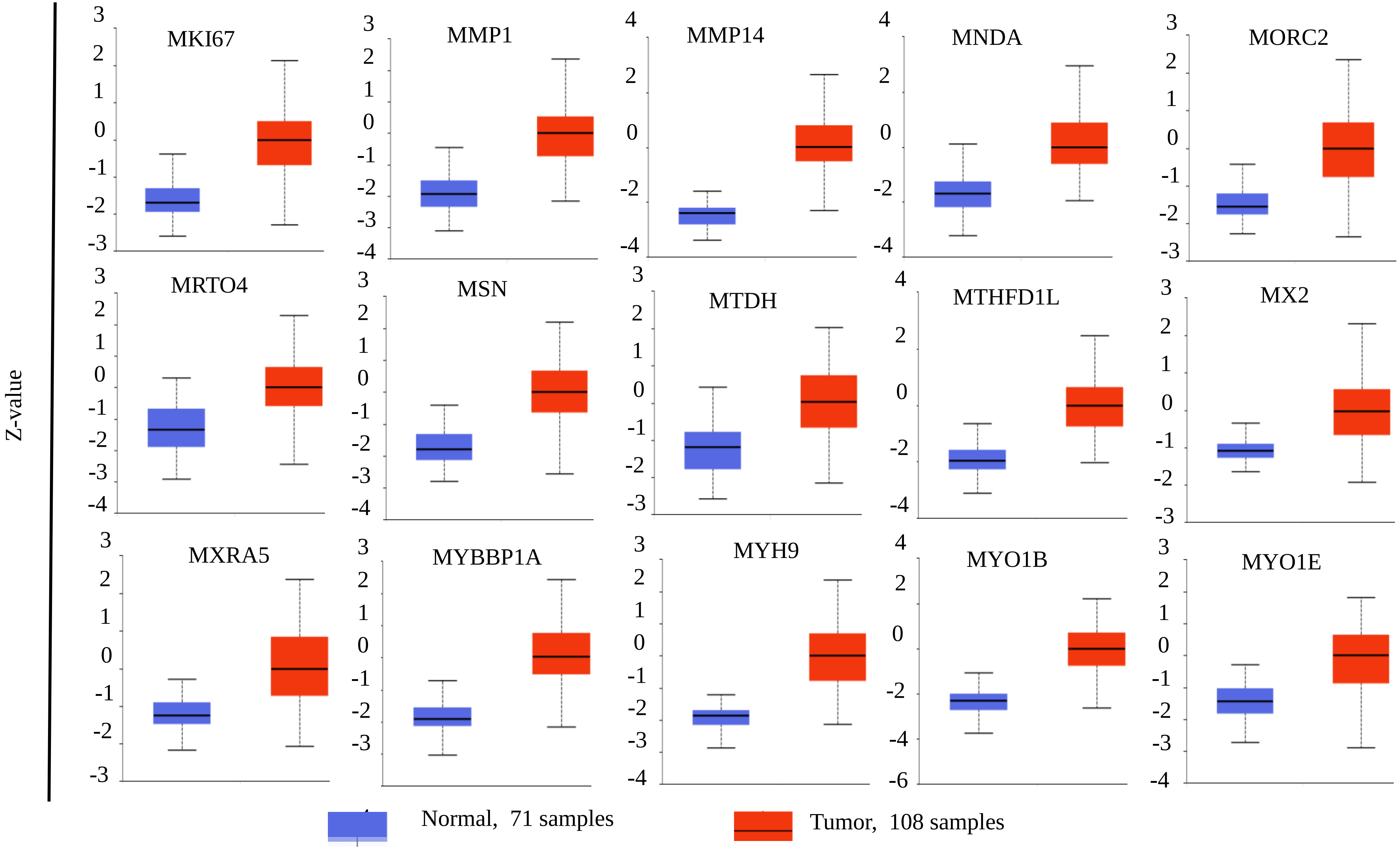

Figure:S5 Protein expression plots of Head and Neck cancer genes. N =Normal sample with respective sample number , T= tumor sample with respective sample number

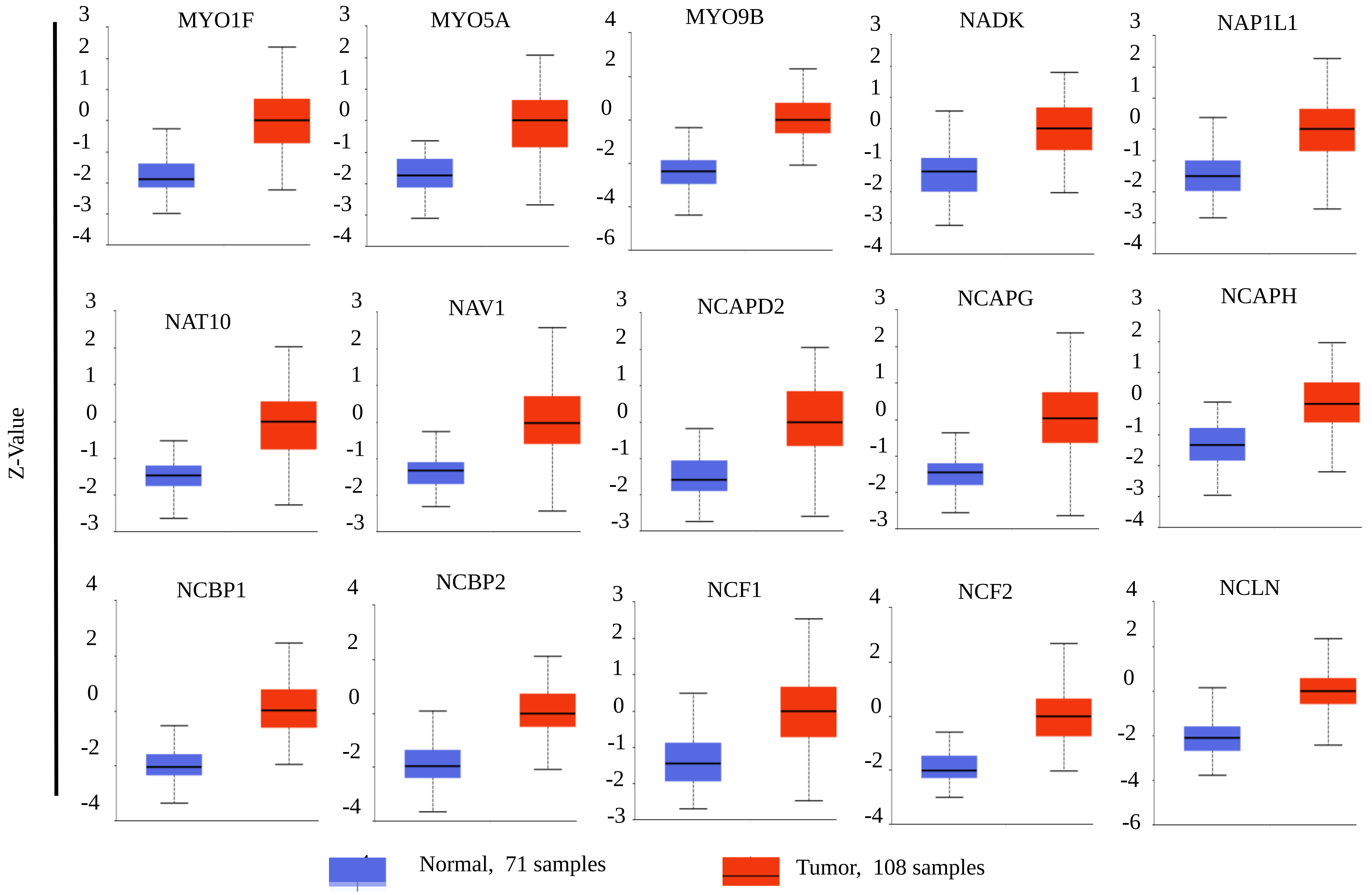

FigureS6: Protein expression plots of Head and Neck cancer genes. N =Normal sample with respective sample number , T= tumor sample with respective sample number

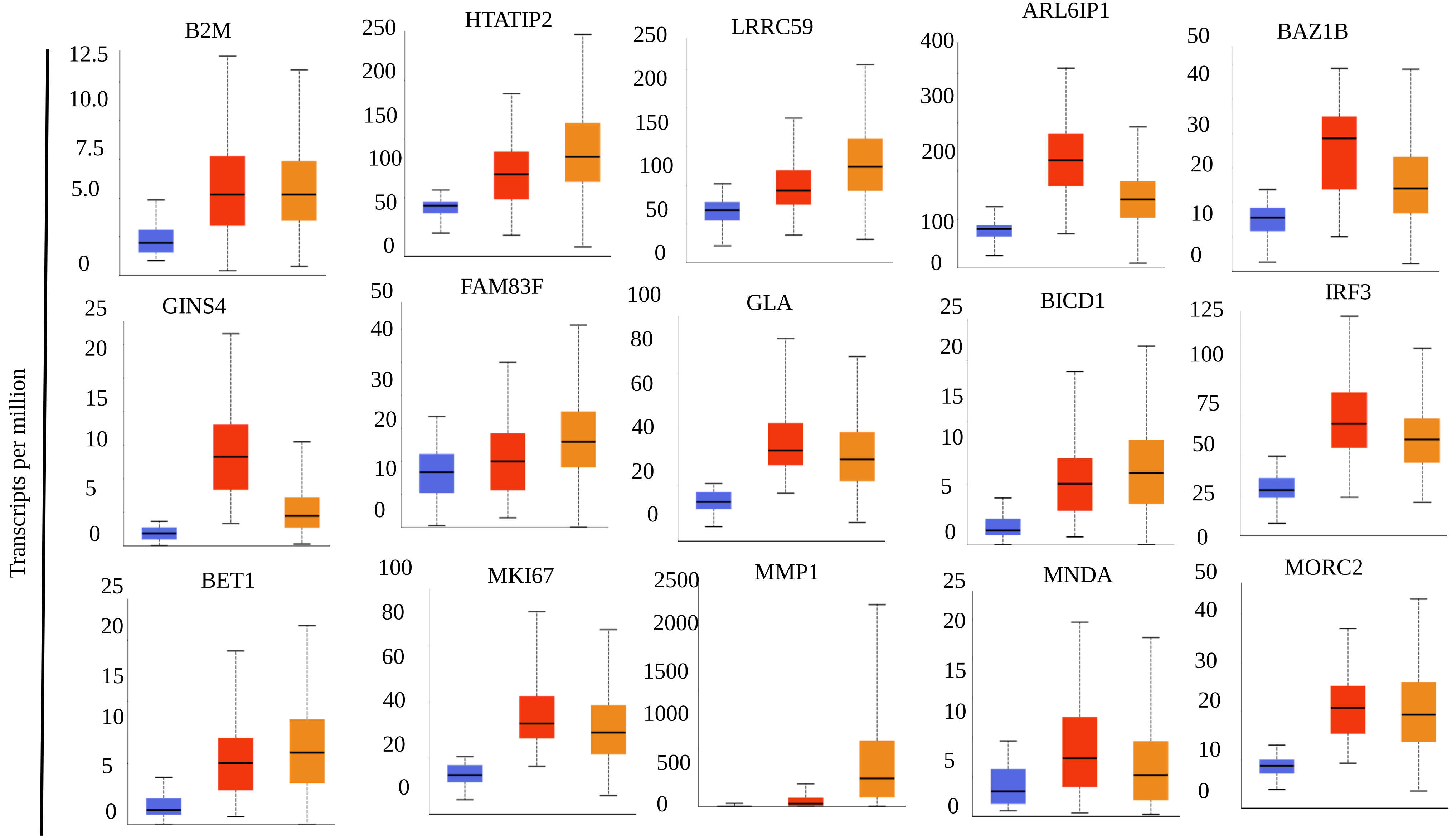

Figure S7: mRNA expression plots of Head and Neck cancer genes with respect to normal, HP positive or negative HNSCC samples.

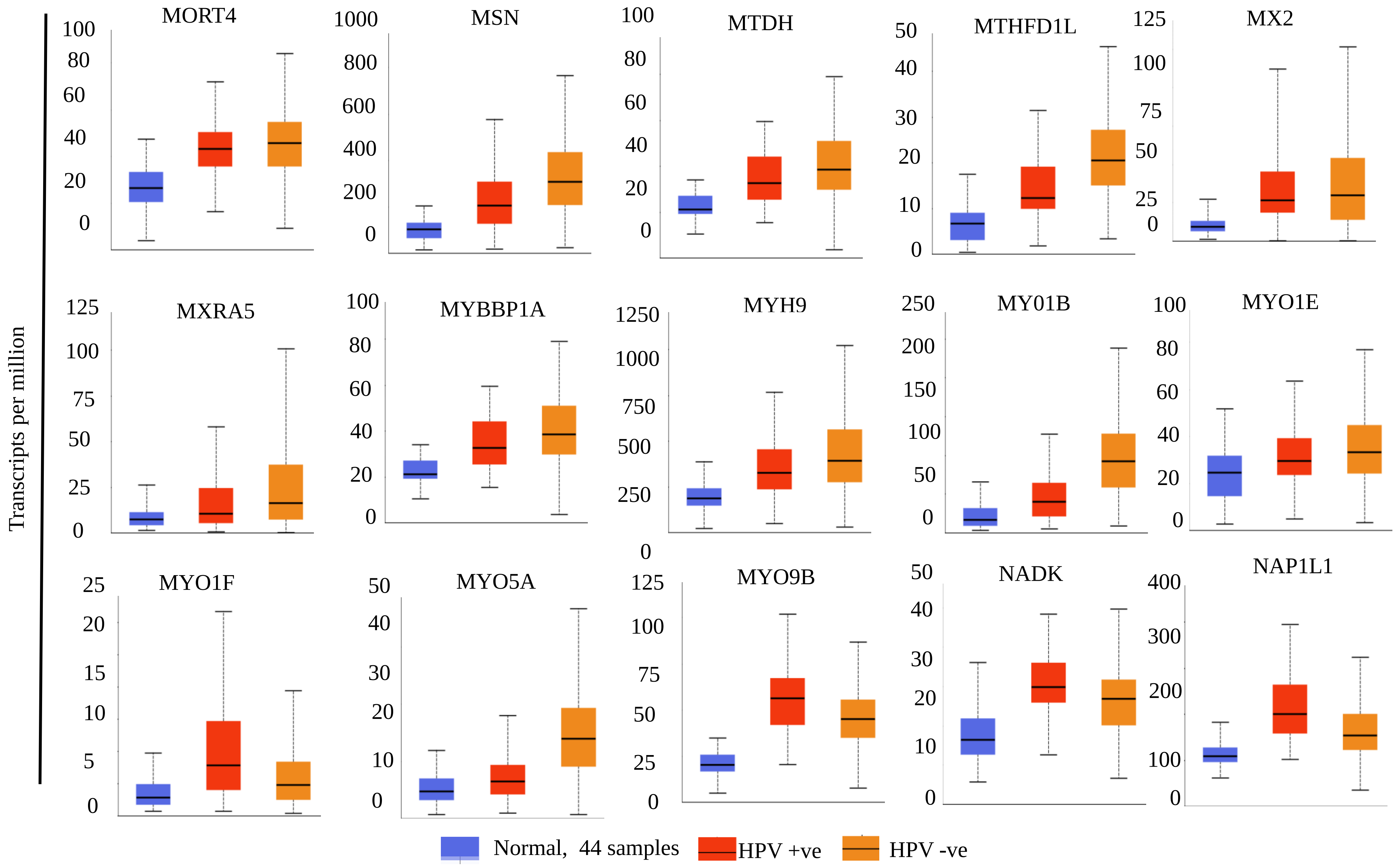

Figure S8: mRNA expression plots of Head and Neck cancer genes with respect to normal, HP positive or negative HNSCC samples.

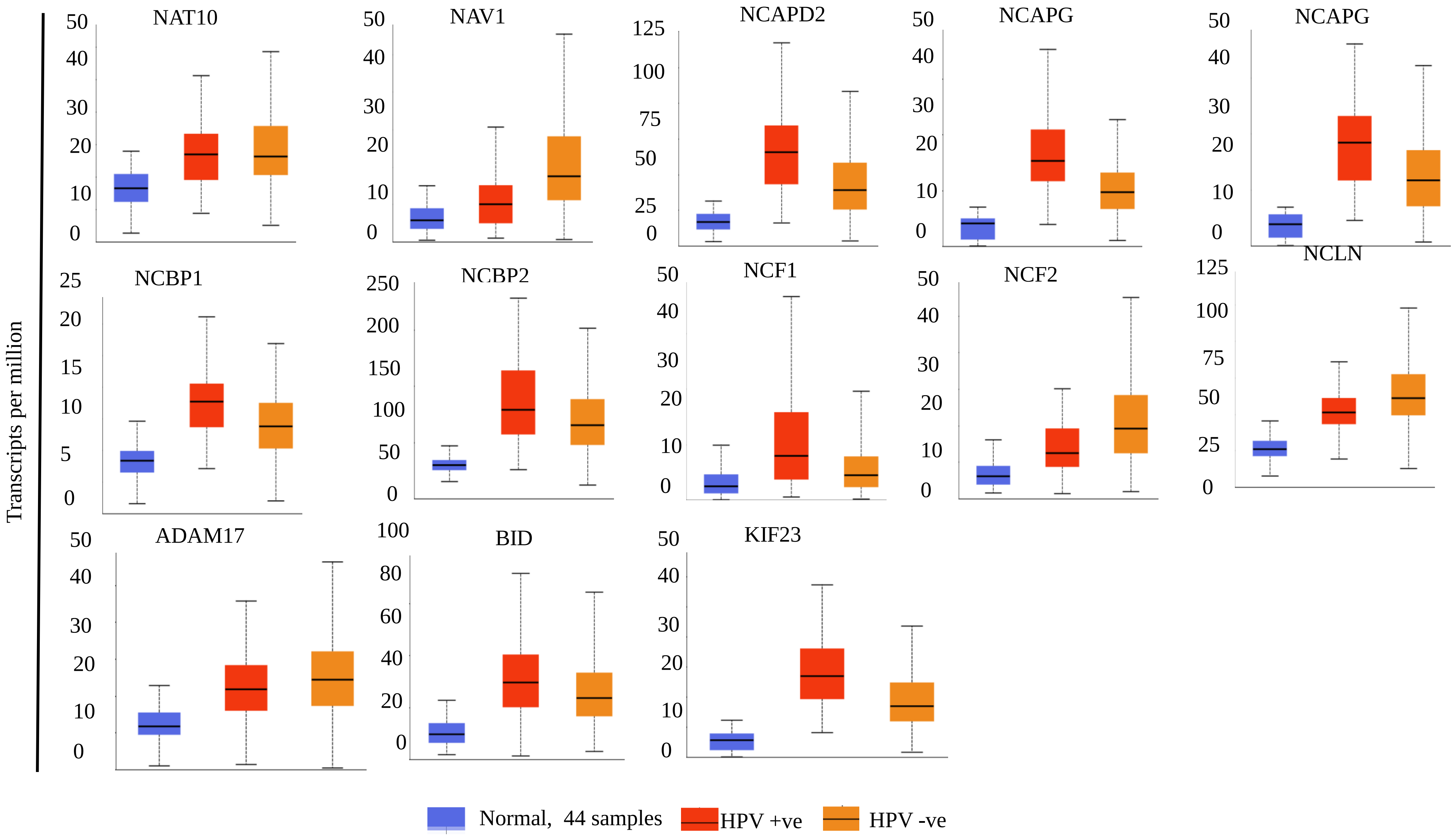

Figure S9: mRNA expression plots of Head and Neck cancer genes with respect to normal, HP positive or negative HNSCC samples.

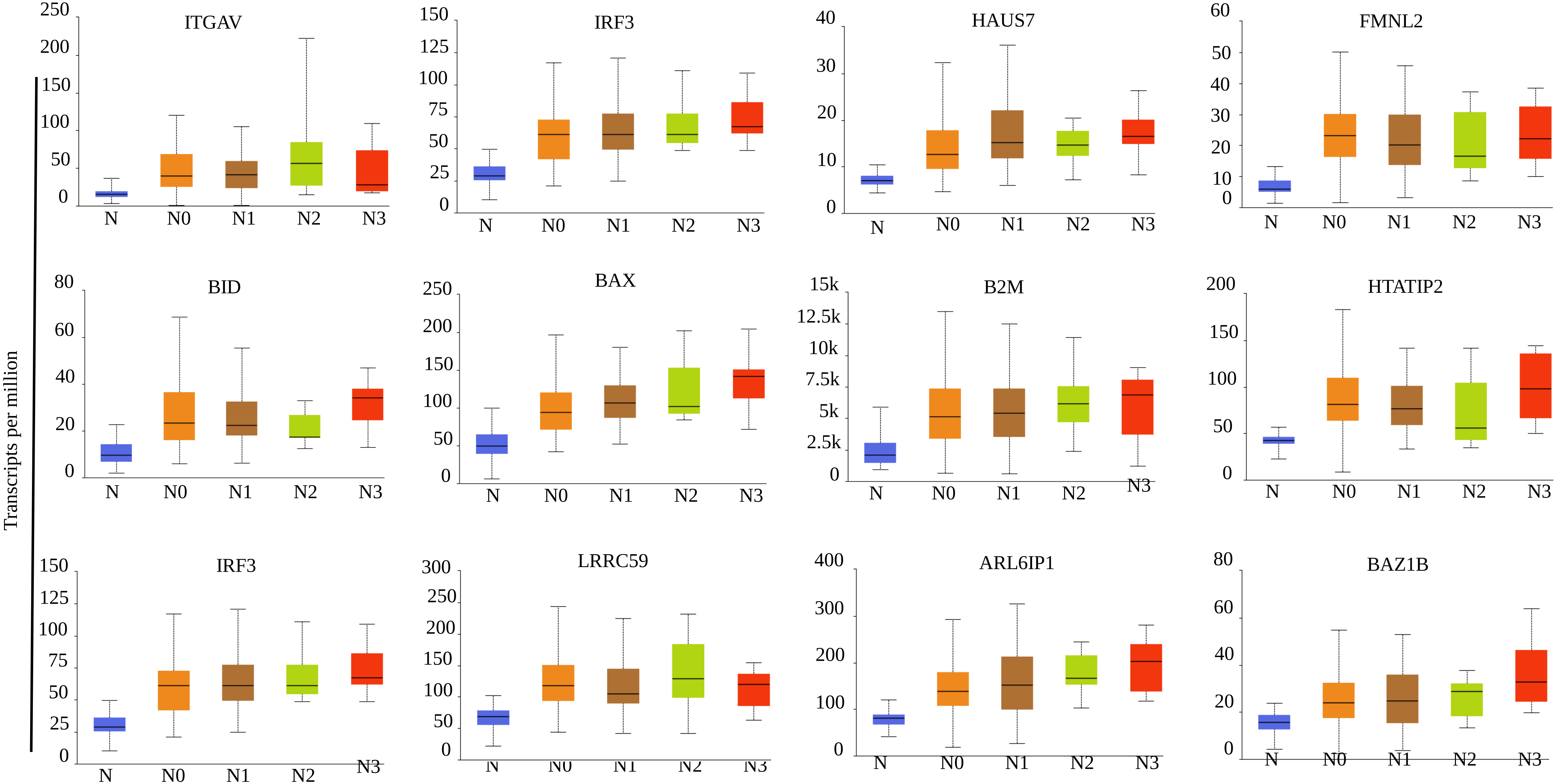

Figure S10: Nodal Metastasis plots of head and neck Cancer genes- N:Normal(samples i.e n=44), N0:No regional lymph node metastasis(n=176), N1:Metastases in 1 to 3 axillary lymph nodes(n=67), N2:Metastases in 4 to 9 axillary nodes(n=12), N3:Metastases in 10 or more axillary lymph nodes(n=8).

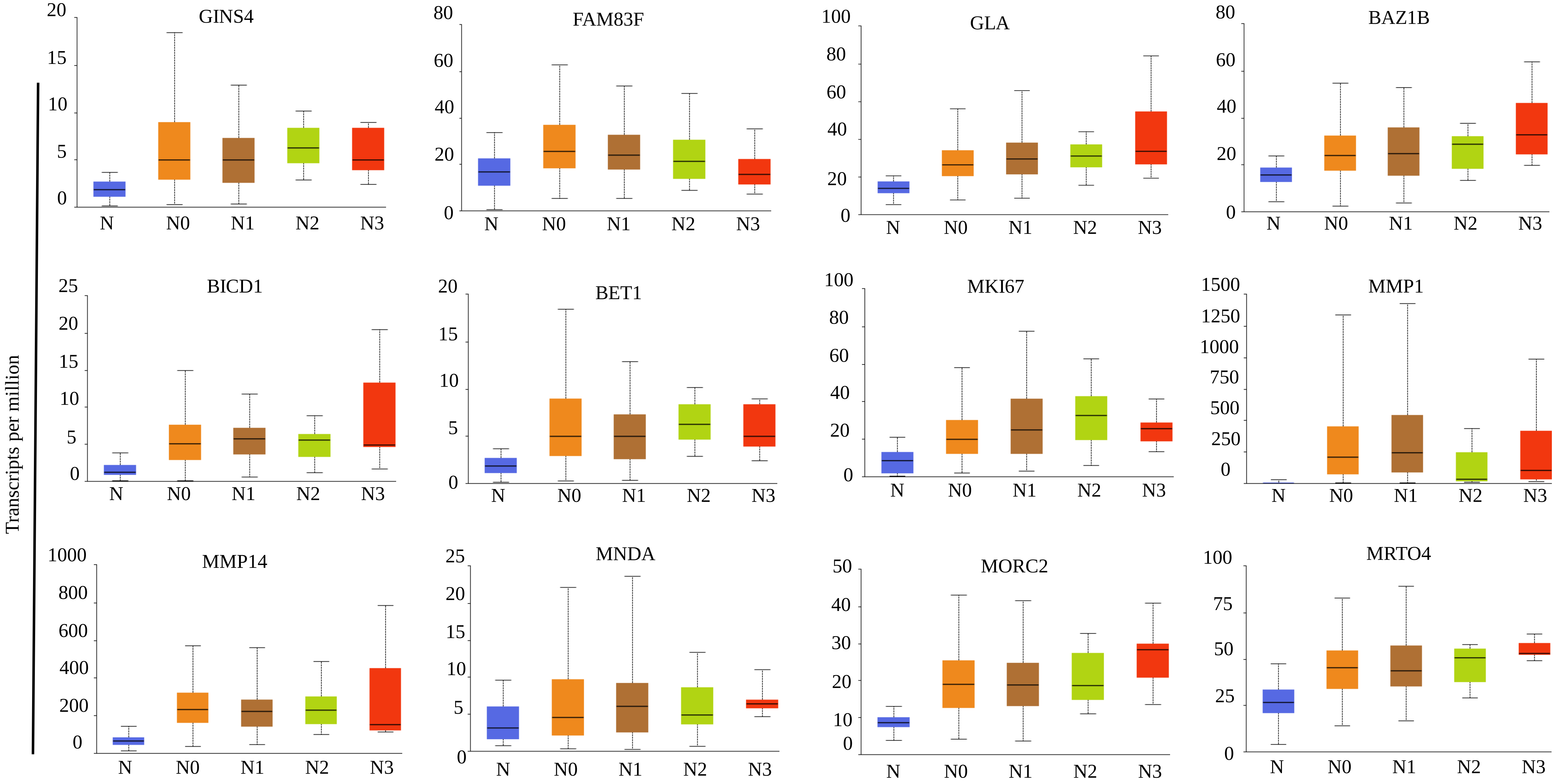

Figure S11: Nodal Metastasis plots of head and neck Cancer genes- N:Normal(samples i.e n=44), N0:No regional lymph node metastasis(n=176), N1:Metastases in 1 to 3 axillary lymph nodes(n=67), N2:Metastases in 4 to 9 axillary nodes(n=12), N3:Metastases in 10 or more axillary lymph nodes(n=8).

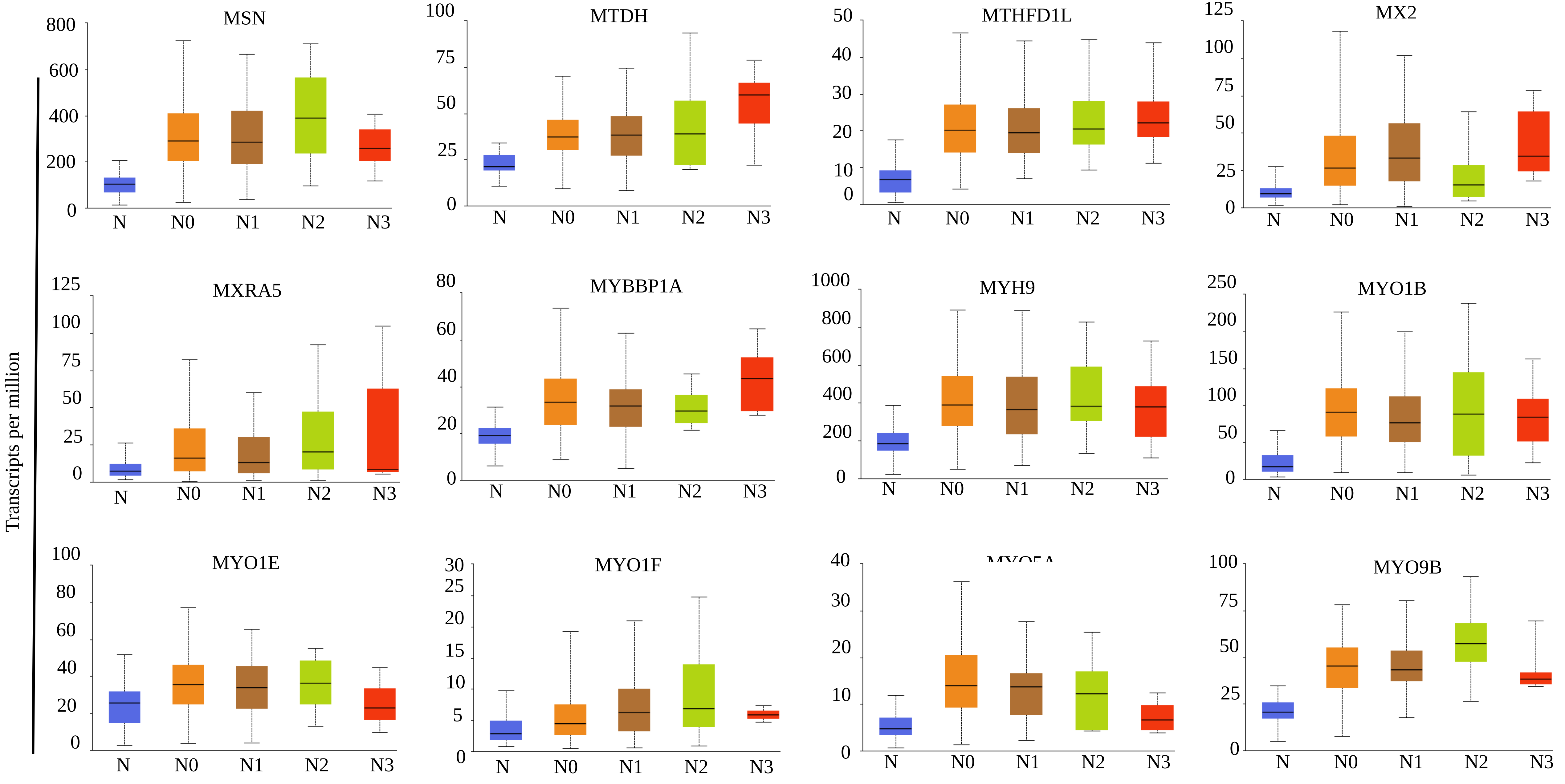

Figure S12: Nodal Metastasis plots of head and neck Cancer genes- N:Normal(samples i.e n=44), N0:No regional lymph node metastasis(n=176), N1:Metastases in 1 to 3 axillary lymph nodes(n=67), N2:Metastases in 4 to 9 axillary nodes(n=12), N3:Metastases in 10 or more axillary lymph nodes(n=8).

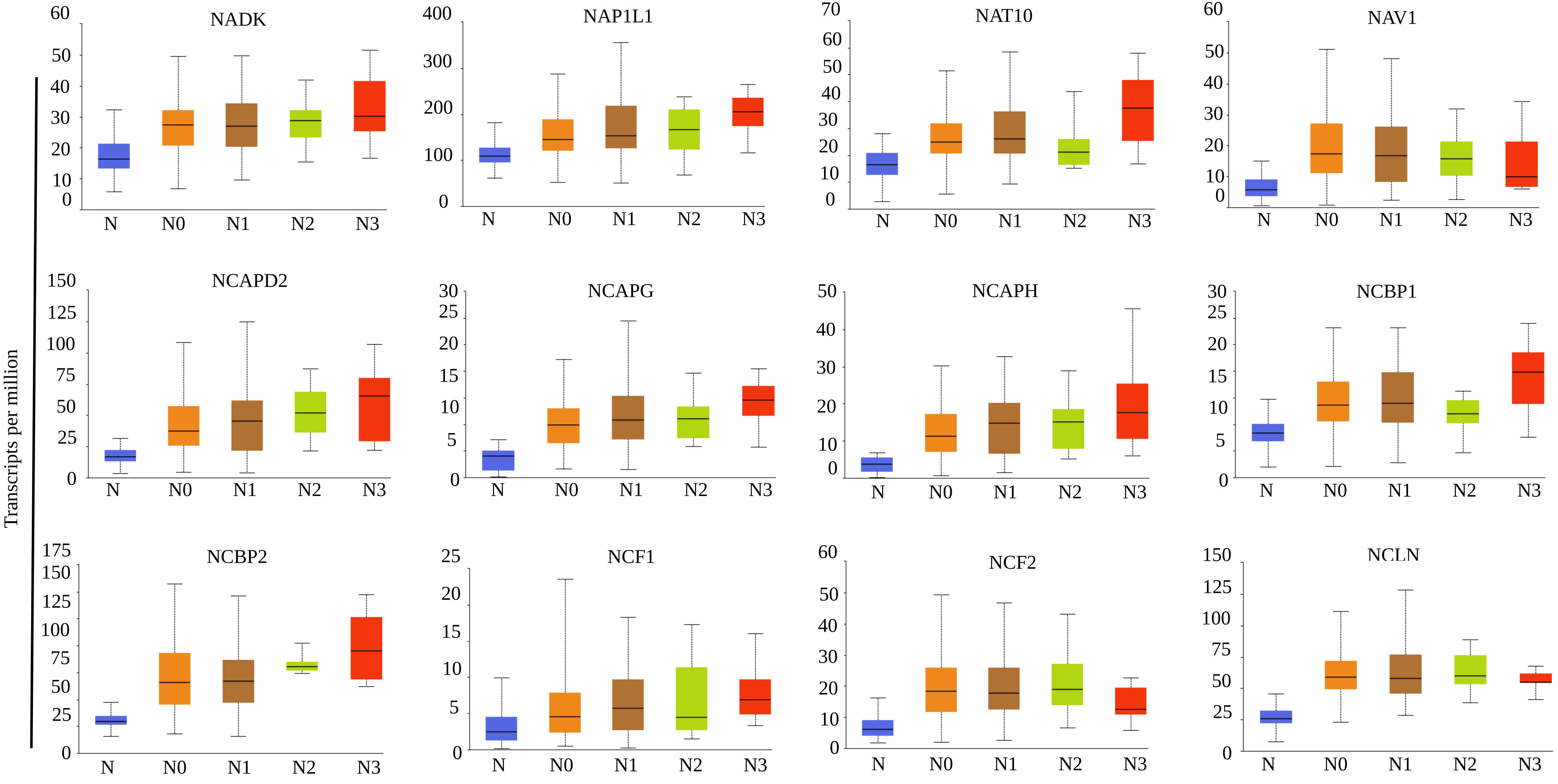

Figure S13: Nodal Metastasis plots of head and neck Cancer genes- N:Normal(samples i.e n=44), N0:No regional lymph node metastasis(n=176), N1:Metastases in 1 to 3 axillary lymph nodes(n=67), N2:Metastases in 4 to 9 axillary nodes(n=12), N3:Metastases in 10 or more axillary lymph nodes(n=8).

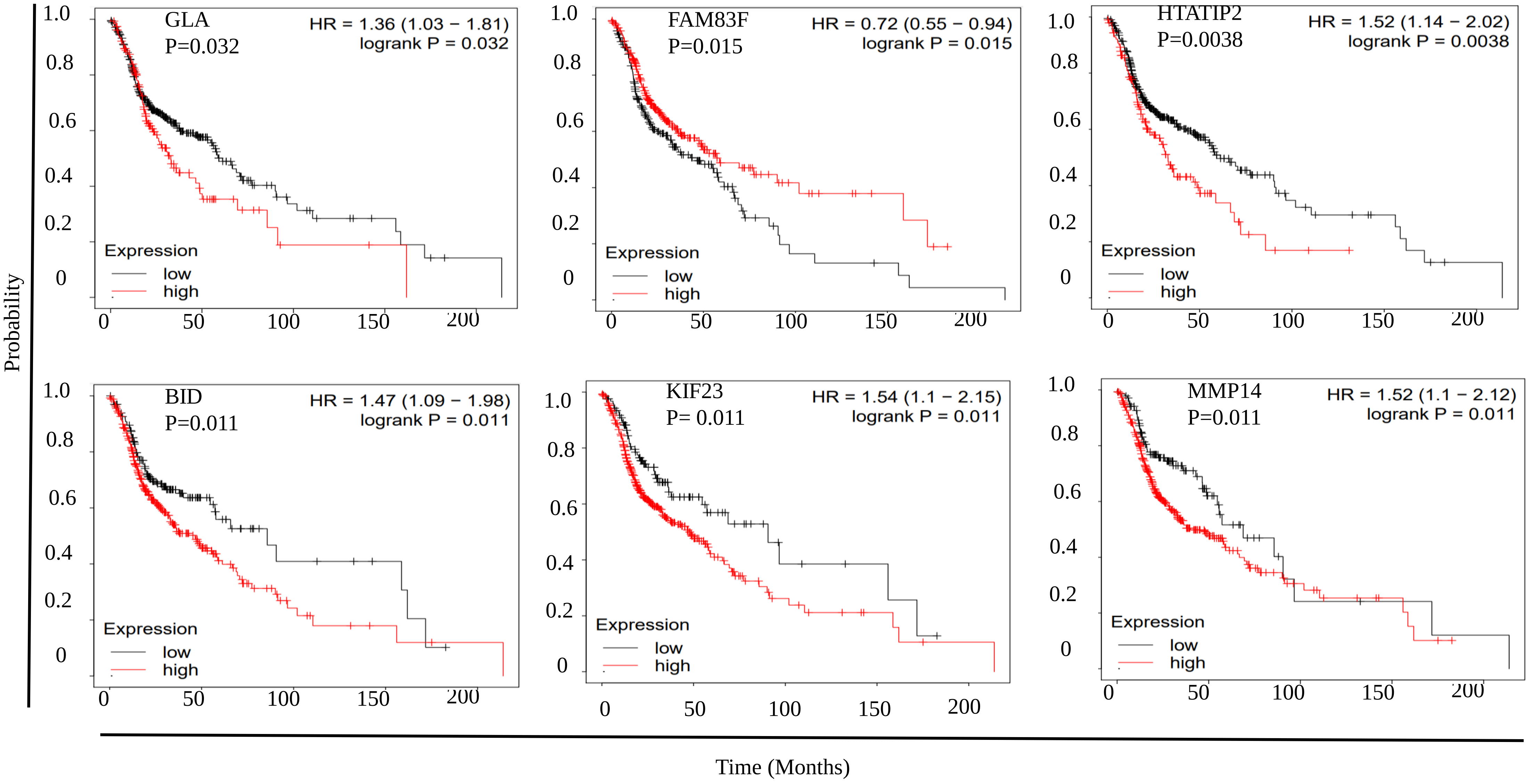

Figure S14: Kaplan –meyer plots for survival based on shortlisted gene expression

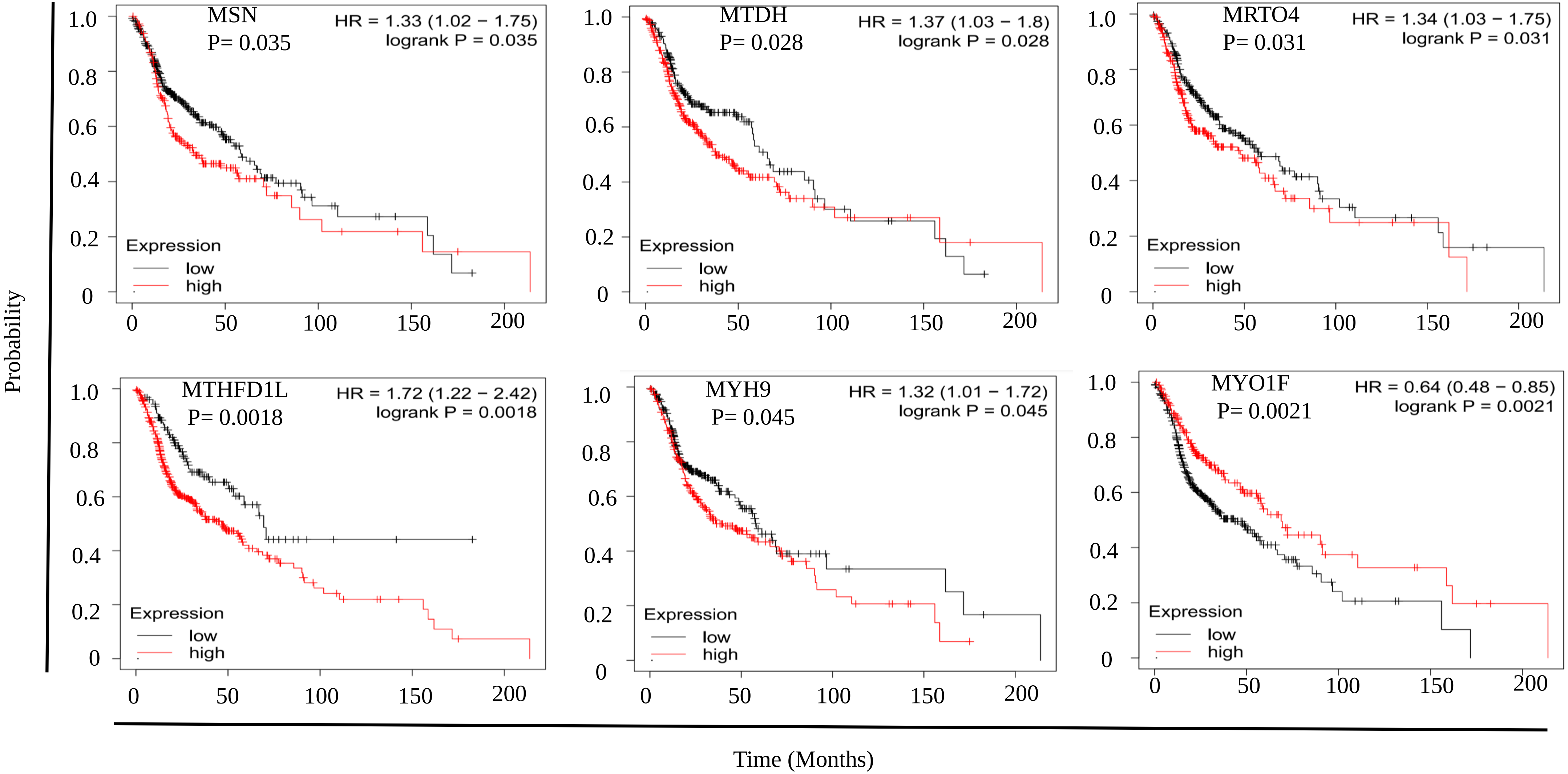

Figure S15: Kaplan –meyer plots for survival based on shortlisted gene expression

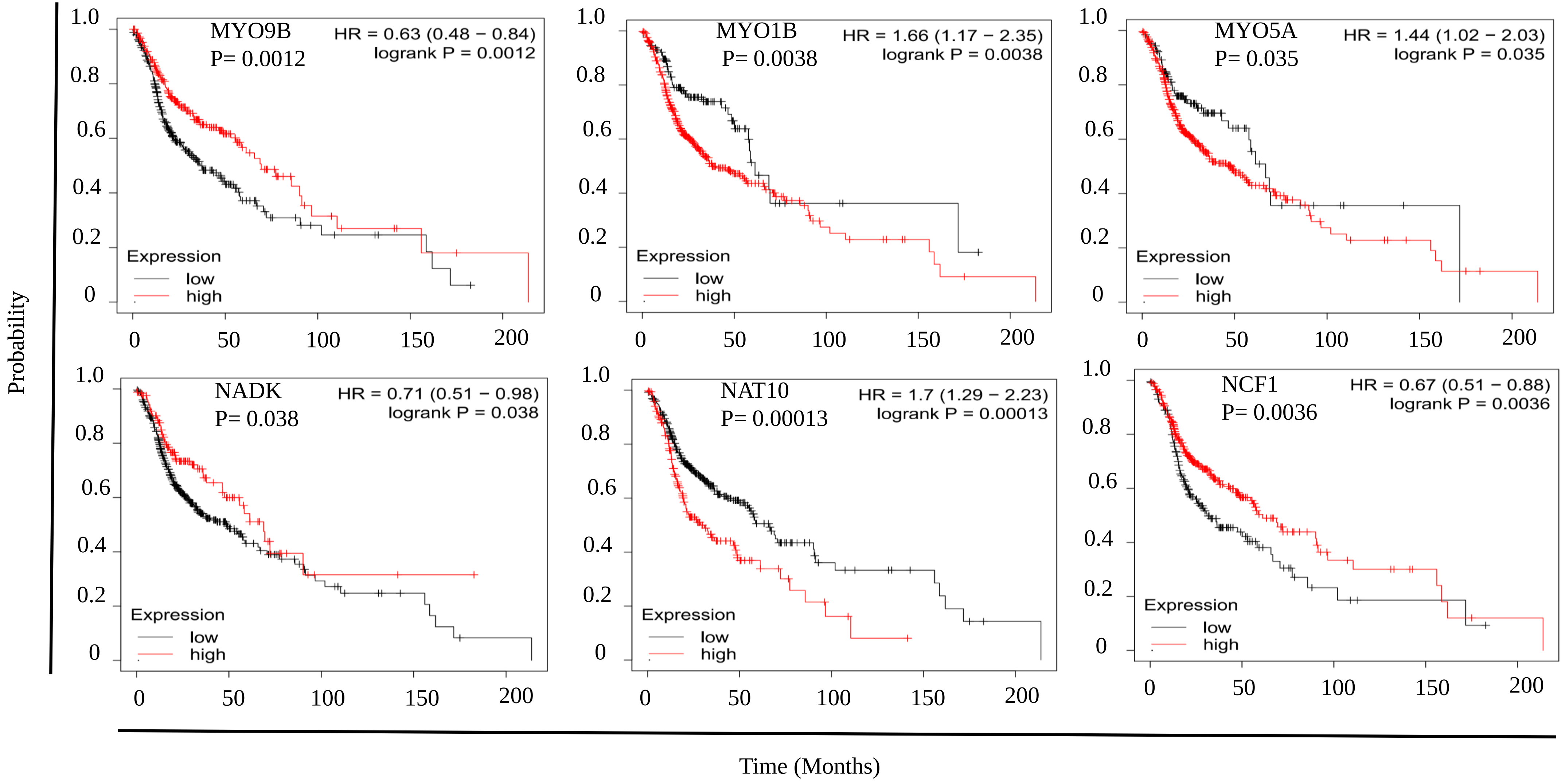

Figure S16: Kaplan –meyer plots for survival based on shortlisted gene expression

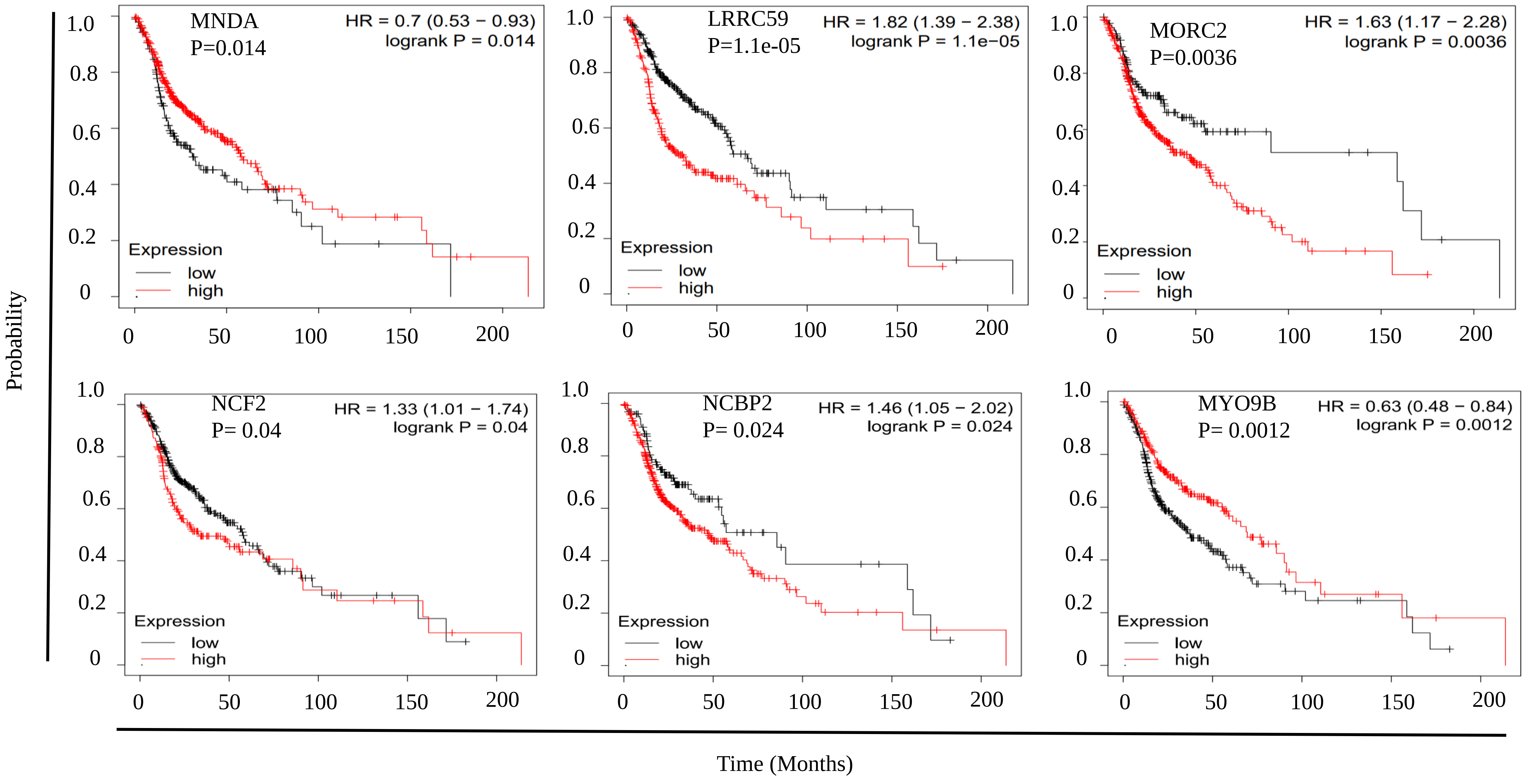

Figure S17: Kaplan –meyer plots for survival based on shortlisted gene expression

Ligand (PubChem SID – 3711570)

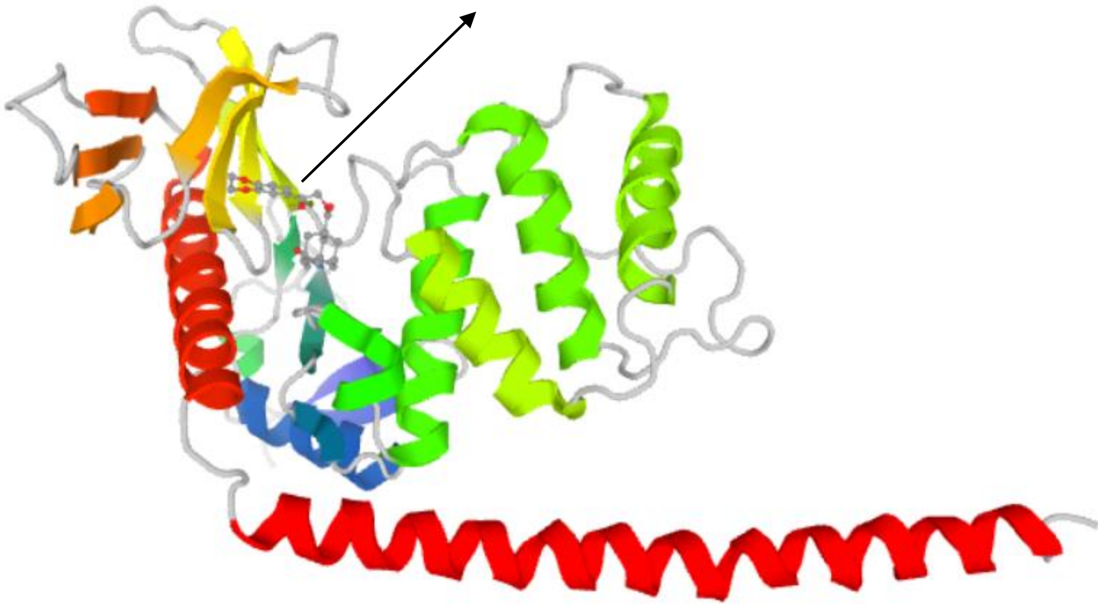

Ligand (PubChem SID – 24840422)

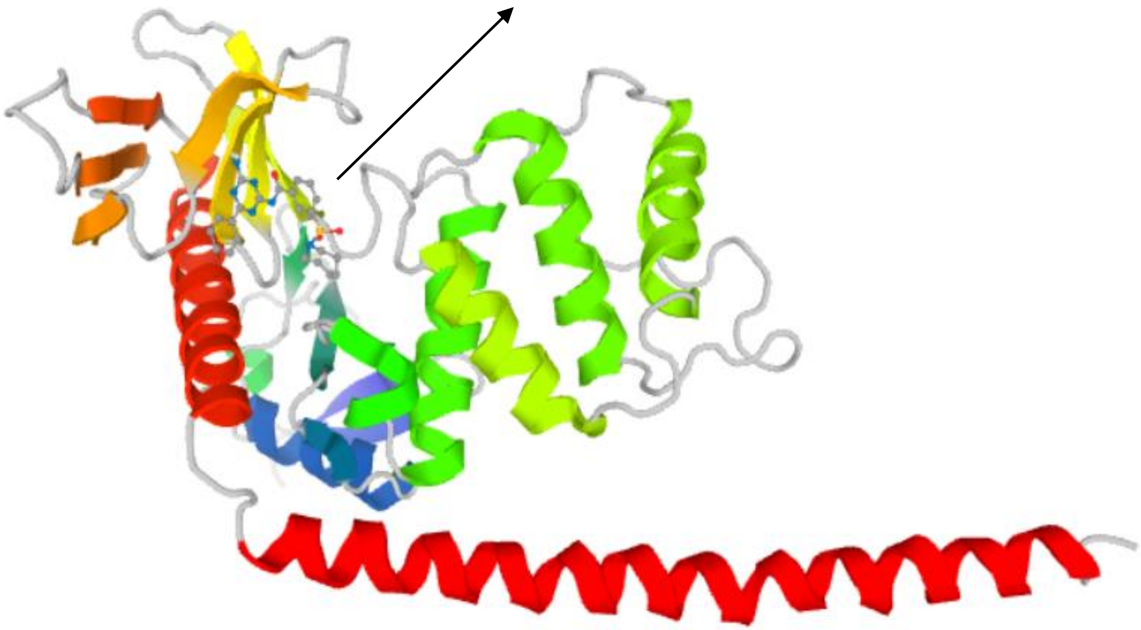

Compound	Model ID	Energy	nRot	HBA	HBD	LogP	MW	TPSA
3711570	1	-8.5	6	7	1	2.4	413.46358	90.93
24840422	1	-8.5	5	9	3	2.81	452.52936	142.78

Figure S18: Binding of ligands to MSN protein. This table represents the ligands with Binding affinity to MSN protein column mentions the Pubchem SID, the Binding Energy is in kCal/mol, nRot- Gives the no of rotatable bonds, HBA- Hydrogen Bond Acceptor, HBD- Hydrogen Bond Donor, LogP is the Partition coefficient, and MW is the Molecular Weight of each compuound. Topological polar surface area (TPSA)- of a molecule is defined as the surface sum over all polar atoms or molecules, primarily oxygen and nitrogen, also including their attached hydrogen atoms.

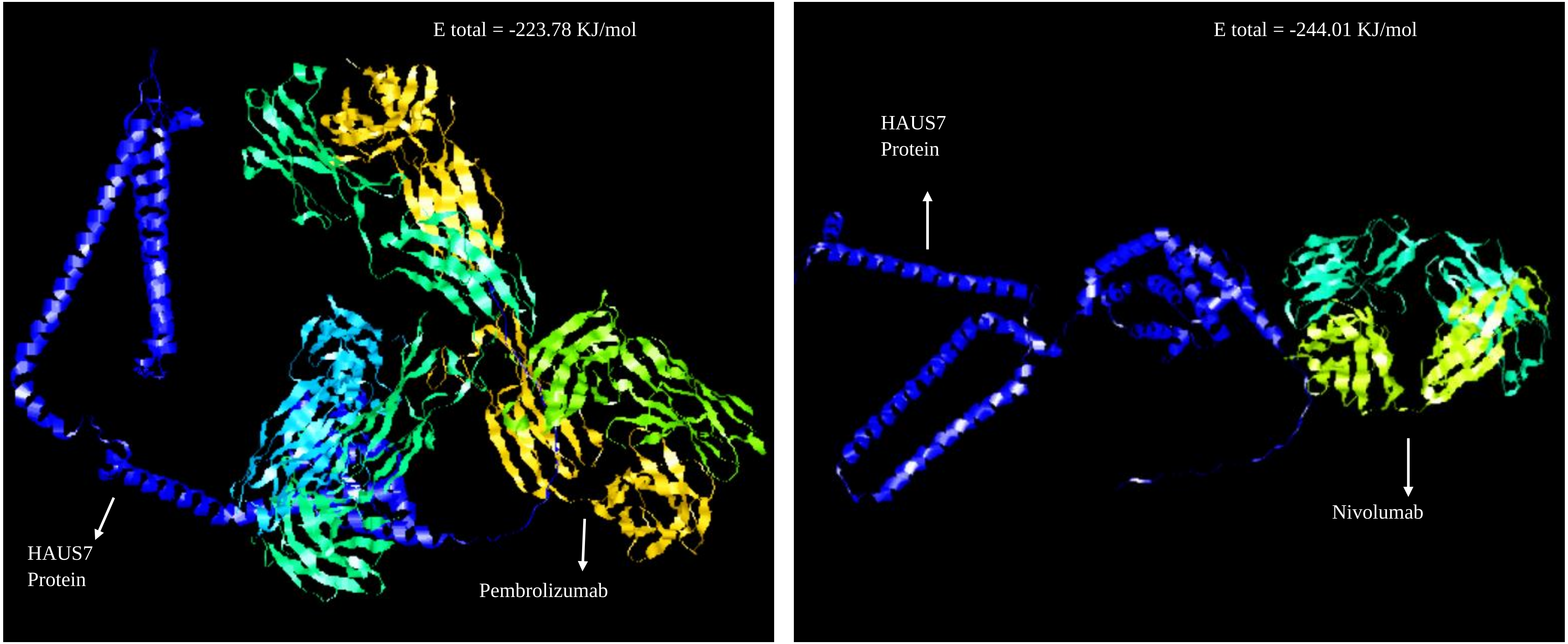

Figure S19: Binding of binding of monoclonal antibodies to HAUS7

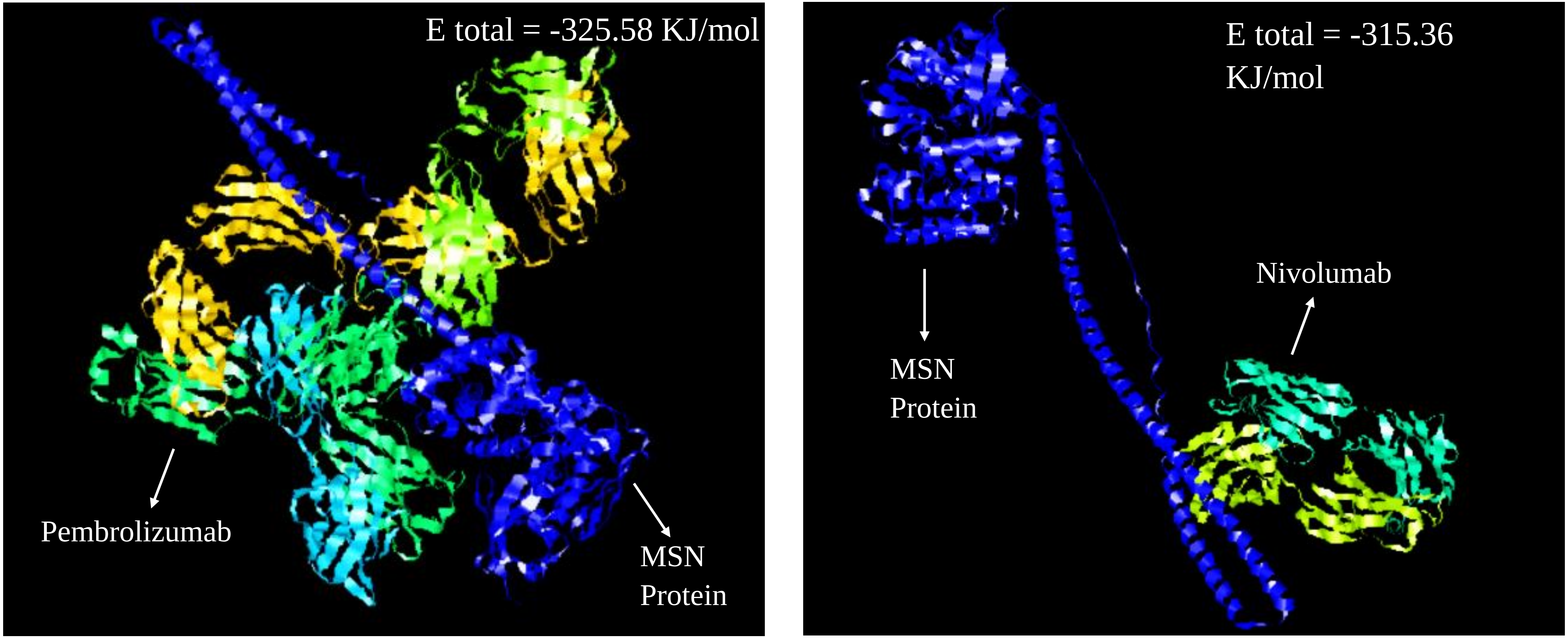

Figure S20: Binding of binding of monoclonal antibodies to MSN

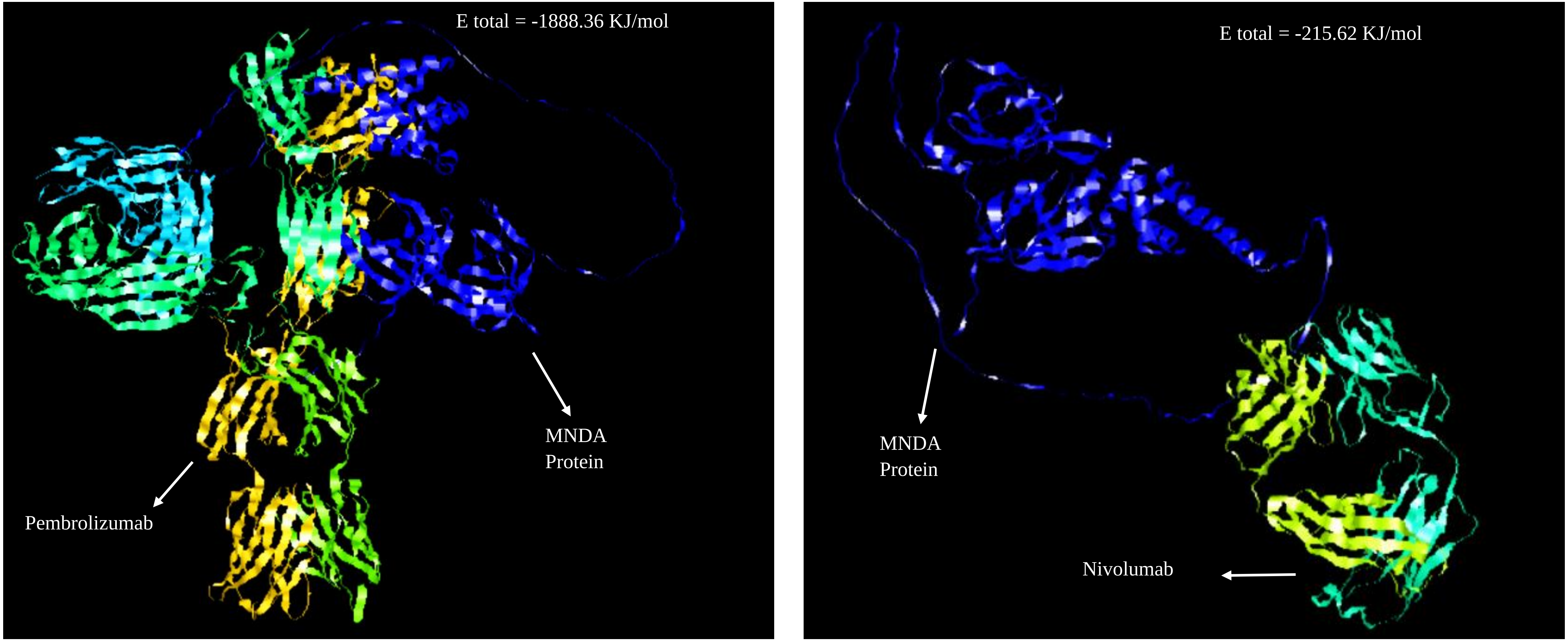

Figure S21: Binding of binding of monoclonal antibodies to MNDA

Genes identified in this study	Was it previously identified in HNC in other Studies	Was it previously identified in any other types of cancers
HAUS7	No	None

Supplementary Table-1: The above table lists out the genes that were identified in this study and is not identified in any other previous research for Head and Neck Cancer or any other type of cancer as well.

Genes identified in this study	Was it previously identified in HNC in other Studies
ARPC1B	Yes
ADAM17	Yes
AMPD3	Yes
MCM7	Yes
LY6E	Yes
KIF23	Yes
ITGAV	Yes
IRF3	Yes
BID	Yes
BAX	Yes
B2M	Yes
HTATIP2	Yes
IRF3	Yes
LRRC59	Yes
GINS4	Yes
GLA	Yes
MKI67	Yes
MMP1	Yes
MMP14	Yes
MORC2	Yes
MRTO4	Yes
MTDH	Yes
MTHFD1L	Yes
MYBBP1A	Yes
MYH9	Yes
MYO1B	Yes
MYO1E	Yes
MYO1F	Yes
MYO5A	Yes
MYO9B	Yes
NAP1L1	Yes
NAT10	Yes
NAV1	Yes
NCAPG	Yes
NCAPH	Yes
NCBP2	Yes
NCF1	Yes
NCF2	Yes
NCLN	Yes

Supplementary Table-2: The above table lists out the genes that were identified in this study and was previously identified in other studies for Head and Neck Cancer as well.
